# The RNF20/40 complex regulates p53-dependent gene transcription and mRNA splicing

**DOI:** 10.1093/jmcb/mjz045

**Published:** 2019-06-01

**Authors:** Chen Wu, Yaqi Cui, Xiuhua Liu, Feng Zhang, Lin-Yu Lu, Xiaochun Yu

**Affiliations:** 1 College of Life Sciences, Hebei University, Baoding 071000, China; 2 Department of Cancer Genetics and Epigenetics, Beckman Research Institute, City of Hope, Duarte, CA 91010, USA; 3 College of Life and Environment Sciences, Shanghai Normal University, Shanghai, China; 4 Key Laboratory of Reproductive Genetics, Ministry of Education and Women’s Reproductive Health Laboratory of Zhejiang Province, Women’s Hospital, School of Medicine, Zhejiang University, Hangzhou, China; 5 Institute of Translational Medicine, Zhejiang University, Hangzhou, China

**Keywords:** RNF20, RNF40, gene transcription, p53, *p21*, *PUMA*

## Abstract

p53 is a key transcription factor to regulate gene transcription. However, the molecular mechanism of chromatin-associated p53 on gene transcription remains elusive. Here, using unbiased protein affinity purification, we found that the RNF20/40 complex associated with p53 on the chromatin. Further analyses indicated that p53 mediated the recruitment of the RNF20/40 complex to p53 target gene loci including *p21* and *PUMA* loci and regulated the transcription of *p21* and *PUMA* via the RNF20/40 complex-dependent histone H2B ubiquitination (ubH2B). Lacking the RNF20/40 complex suppressed not only ubH2B but also the generation of the mature mRNA of *p21* and *PUMA*. Moreover, ubH2B was recognized by the ubiquitin-binding motif of pre-mRNA processing splicing factor 8 (PRPF8), a subunit in the spliceosome, and PRPF8 was required for the maturation of the mRNA of *p21* and *PUMA*. Our study unveils a novel p53-dependent pathway that regulates mRNA splicing for tumor suppression.

## Introduction

p53 is an important tumor suppressor that is often mutated in human cancer. It maintains genomic stability and governs numerous biological processes. One key function of p53 is regulating gene transcription in response to genotoxic stress, which results in cell cycle arrest, apoptosis, senescence, etc. (Beckerman and Prives, 2010; Bieging et al., 2014; Joerger and Fersht, 2016).

The transcription activity of p53 is mediated by its unique domain architecture. p53 has five major domains, namely an N-terminal transactivation domain (TAD), a proline-rich region (PR), a DNA-binding domain (DBD), a tetramerization domain (TET), and a C-terminal regulatory domain (CTD; Beckerman and Prives, 2010). The key domain that participates in transcription regulation is the DBD (Beckerman and Prives, 2010). In response to genotoxic stress, the expression of p53 mRNA and protein is remarkably elevated (Murray-Zmijewski et al., 2008; Vousden and Prives, 2009). p53 is stabilized and recognizes the sequence specific response element (RE) in the promoter regions of its targeting genes via the DBD for gene transcription (Hainaut and Milner, 1993; McLure and Lee, 1998; Kitayner et al., 2006). To date, most cancer-associated mutations occur in the DBD domain (Vogelstein and Kinzler, 1992; Olivier et al., 2002). Loss of the DBD abolishes p53-dependent gene transcription (Shay et al., 1992; Liu and Bodmer, 2006). In addition to the DBD, the CTD is also reported to facilitate DNA binding as several lysine residues in this domain provide positive charges for nucleic acid binding (McKinney et al., 2004; Liu and Kulesz-Martin, 2006). Posttranslational modifications including acetylation and methylation on these lysine residues in the CTD also regulate the DNA binding as well as gene transcription (Avantaggiati et al., 1997; Gu and Roeder, 1997; Sakaguchi et al., 1998; Chuikov et al., 2004).

The p53 RE is evolutionarily conserved in genes that regulate cell cycle checkpoints (el-Deiry et al., 1992; Funk et al., 1992). The typical example is the *p21* gene that contains two REs at the promoter regions. Once p53 recognizes RE, it mediates the recruitment of chromatin remodeling complex, such as p300/CBP, pCAF, GCN5, and TIP60, to establish chromatin condition for loading the preinitiation complex for RNA pol II-dependent transcription (Avantaggiati et al., 1997; Candau et al., 1997; Gu and Roeder, 1997; Lill et al., 1997; Scolnick et al., 1997; Barlev et al., 2001; Gevry et al., 2007).

In addition to transcription initiation, accumulated evidence shows that p53 plays a very important role in transcription elongation and pre-mRNA splicing (Balakrishnan and Gross, 2008; Dutertre et al., 2011). Once RNA pol II proceeds transcription, p53 associates with several elongation factors including cdk9, the FACT complex, ELL, and the MED1 complex to modulate the gene body region for RNA pol II-dependent transcription elongation (Gu et al., 1999; Shinobu et al., 1999; Keller et al., 2001; Zhang et al., 2005; Claudio et al., 2006; Radhakrishnan and Gartel, 2006). Moreover, disruption of the pre-mRNA splicing is able to activate p53 (Allende-Vega et al., 2013). However, the detailed molecular mechanism of p53-dependent transcription elongation and pre-mRNA splicing remains elusive.

Similar to p53, other chromatin remodelers are also required for transcription elongation. One important chromatin remodeler for transcription elongation is the RNF20/40 complex (Xiao et al., 2005; Zhu et al., 2005; Pavri et al., 2006; Kim et al., 2009). The RNF20/40 complex is evolutionarily conserved in eukaryotes (Hwang et al., 2003; Wood et al., 2003) and consists of RNF20 and RNF40 (Kim et al., 2005; Zhu et al., 2005). Both RNF20 and RNF40 contain a Ring domain at the C-terminus and act as ubiquitin E3 ligase for histone H2B ubiquitination (ubH2B; Kim et al., 2005; Zhu et al., 2005). In addition, the complex directly interacts with WAC, an adaptor to link RNF20 and RNF40 with RNA pol II (Zhang and Yu, 2011). During transcription elongation, the RNF20/40 complex ubiquitinates H2B, which facilitates the removal of histone barrier for transcription elongation (Minsky et al., 2008; Shema et al., 2008). Moreover, the complex suppresses the interaction between TFIIS and the PAF complex (Shema et al., 2011) and acts as the tumor suppressor to target a set of gene transcription (Shema et al., 2008). However, the mechanism by which the RNF20/40 complex is recruited for transcription regulation remains elusive.

Here, using unbiased protein affinity purification, we found that p53 interacts with the RNF20/40 complex in the chromatin fraction. This interaction mediates the recruitment of the RNF20/40 complex for ubH2B at the gene body region of *p21* and *PUMA*. Moreover, the RNF20/40 complex-dependent ubH2B not only mediates the transcription elongation but also facilitates mRNA splicing by recruiting pre-mRNA processing splicing factor 8 (PRPF8), a key subunit in the spliceosome. Thus, our studies reveal an important molecular mechanism underlying transcription elongation and mRNA splicing.

## Results

### p53 is a binding partner of RNF20/40

p53 is a transcription factor and binds to genomic DNA following DNA damage. Moreover, it is also a chromatin-associated protein. To examine the chromatin-associated p53, we treated cells with 20 J/m^2^ UV-C. The cells were lyzed with NETN100 buffer (0.5% NP-40, 50 mM Tris–HCl [pH 8.0], 2 mM ethylenediaminetetraacetic acid [EDTA], and 100 mM NaCl), and the insoluble chromatin fraction was further treated with high-salt NETN300 buffer (0.5% NP-40, 50 mM Tris–HCl [pH 8.0], 2 mM EDTA, and 300 mM NaCl). The elutes with chromatin-bound proteins were harvested, and we verified that p53 was existed in this fraction (Supplementary Figure S1). In particular, in response to DNA damage, substantial amount of p53 was expressed and associated with genomic DNA (Supplementary Figure S1).

**Figure 1:**
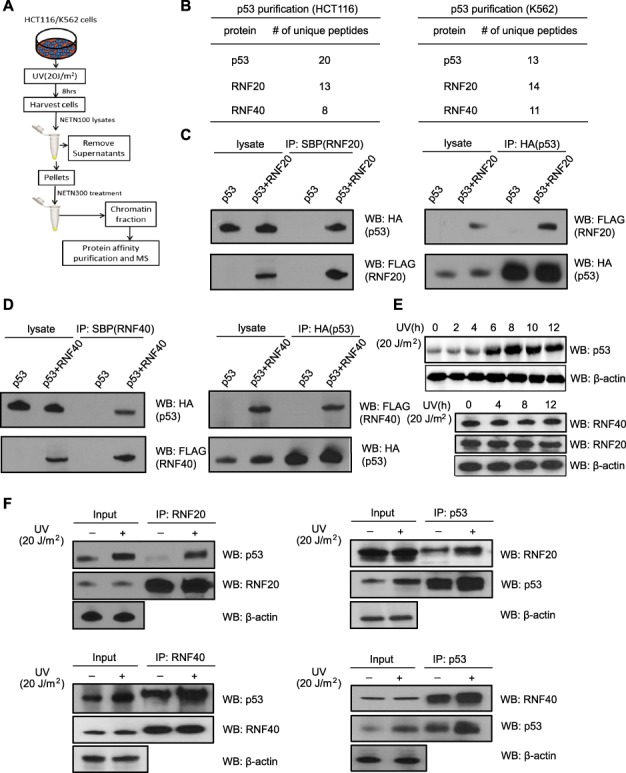
p53 associates with the RNF20/40 complex. (**A**) The sketch of searching for the DNA damage-induced p53 complex on the chromatin by protein affinity purification and MS. Chromatin fractions isolated from HCT116 and K562 cells stably expressing SFB-p53 were subjected to affinity purification. Eluted proteins were examined by MS. (**B**) The list of peptides identified by p53 affinity purification and MS. (**C** and **D**) p53 interacts with RNF20 and RNF40. HCT116 cells were transfected with vectors encoding HA-p53 and SFB-RNF20 (**C**) or RNF40 (**D**). Cells were lyzed with NETN300 buffer. Cell extracts were examined by IP and western blot with indicated antibodies. (**E**) Endogenous expression of p53, RNF20, and RNF40 in HCT116 cells were examined at indicated time points following UV-C (20 J/m^2^). (**F**) p53 associates with endogenous RNF20/40. HCT116 cells were treated with UV-C (20 J/m^2^). After 8 h recovery, cells were lyzed with NETN300 buffer, and lysates were examined by IP and western blot with indicated antibodies. β-actin was used as the protein-loading control.

In order to identify the chromatin-associated functional partner(s) of p53, we performed unbiased tandem affinity purification using p53 as the bait. The full-length p53 was stably expressed in HCT116 and K562 cells. As wild-type p53 is expressed in both HCT116 and K562 cells, we compared and found the expression level of exogenous p53 was less than that of the endogenous p53 (Supplementary Figure S2). We harvested the chromatin fraction using the same protocol as discussed above and performed affinity protein purification of p53 using SBP tag and S tag. The chromatin fraction was incubated with streptavidin-conjugated agarose, which was eluted with excessive biotin. The eluted proteins were then incubated with S beads, and the bound proteins were examined by mass spectrometry (MS; Figure 1A). Our proteomic analysis indicates that p53 is associated with the RNF20/40 complex (aka BRE1A and BRE1B) in both HCT116 and K562 cells (Figure 1B; Supplementary Tables S1 and S2). Interestingly, the RNF20/40 complex is an E3 ligase complex that mediates histone H2B ubiquitination and regulates gene transcription elongation (Kim et al., 2005; Zhu et al., 2005). In addition to the RNF20/40 complex, we also detected CUL9 (aka PARC), CUL7, and FBXW8 in the p53 purifications, which are known p53-associated proteins that may regulate p53 biogenesis and its functions (Nikolaev et al., 2003; Andrews et al., 2006; Skaar et al., 2007).

### Mapping the interaction domains in p53 and RNF20/40

To validate the interaction between p53 and the RNF20/40 complex, we performed co-immunoprecipitation (co-IP) and western blot assays in HCT116 cells and found that, in the chromatin fraction, p53 was co-IPed with the RNF20/40 complex and *vice versa* (Figure 1C and D; Supplementary Figure S3A and B). Moreover, we examined the endogenous interaction between p53 and the RNF20/40 complex in HCT116 cells. When p53 reached the peak level at 8 h after UV treatment (Figure 1E), the interaction between p53 and the RNF20/40 complex was remarkably increased (Figure 1F; Supplementary Figure S3C and D). Meanwhile, we did not find any changes in the expression of the RNF20/40 complex following DNA damage (Figure 1E). Moreover, we also treated HCT116 cells with methyl methanesulfonate (MMS; 2 mM), an alkylating agent with DNA-damaging effects, and found that the RNF20/40 complex was associated with p53 in HCT116 cells expressing wild-type p53 (Supplementary Figure S3E). Thus, our results demonstrate that following DNA damage, upregulated p53 associates with the RNF20/40 complex.

**Figure 2:**
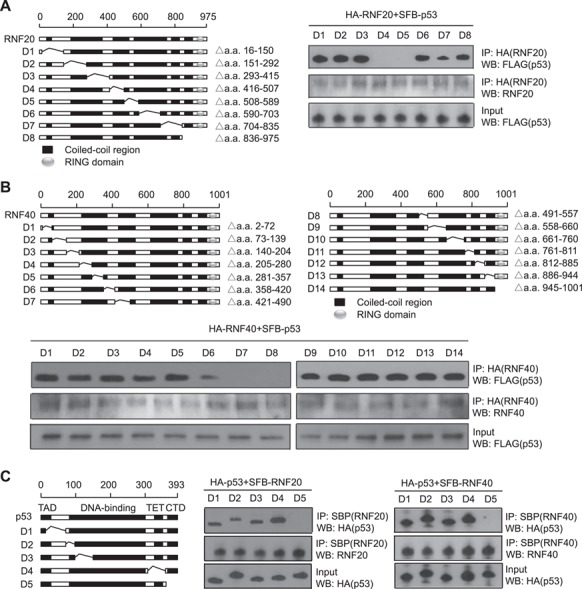
Mapping the interaction domains in p53 and RNF20/40. (**A** and **B**) The third coiled-coil regions of RNF20 or RNF40 mediated the interactions with p53. The internal deletion mutants of HA-tagged RNF20 or RNF40 and SFB-p53 were expressed in 293T cells. Cell lysates were examined by co-IP and western blots with the indicated antibodies. (**C**) The CTD of p53 is required for the interaction with RNF20/40. HA-tagged p53 internal deletion mutants and SFB-RNF 20 or SFB-RNF40 were expressed in 293T cells. The interaction between RNF20/40 and p53 were examined by co-IP and western blots with the indicated antibodies.

Next, we mapped the interaction regions on both p53 and the RNF20/40 complex. Both RNF20 and 40 have multiple coiled-coil regions and a C-terminal Ring domain. Using the internal deletion mutants, we found that the third coiled-coil region in either RNF20 or RNF40 mediated the interactions with p53 (Figure 2A and B). Moreover, with the same approach, we found that the CTD of p53 is required for the interaction with RNF20/40 (Figure 2C).

### p53 is recruited to the *p21* gene locus in response to DNA damage

**Figure 3:**
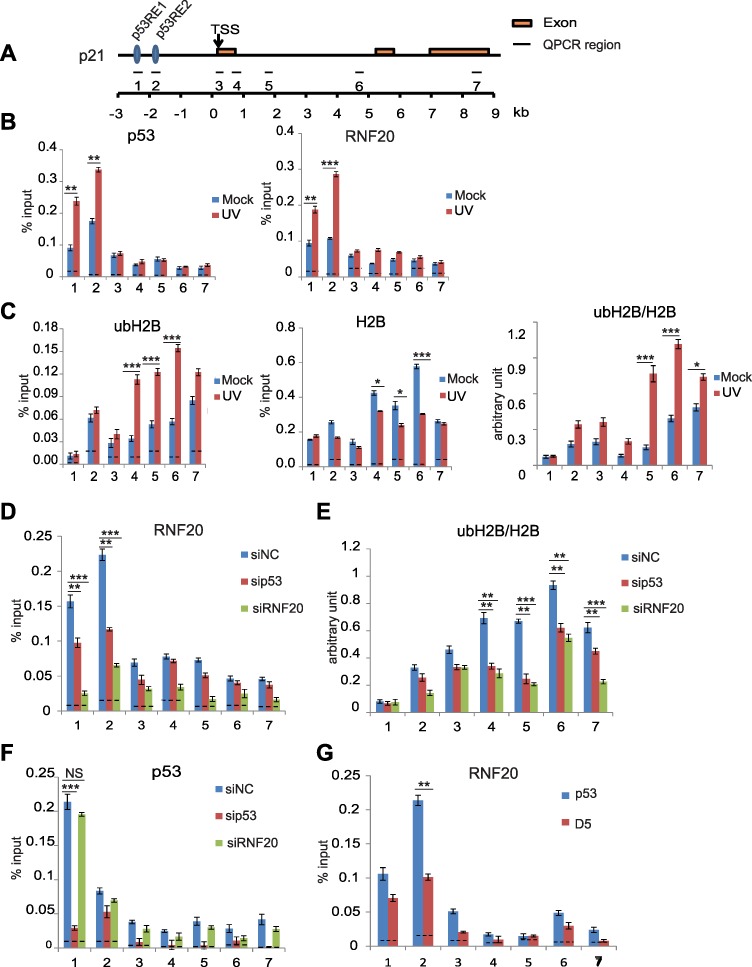
p53 mediates the recruitment of the RNF20/40 complex to the promoter region of *p21*. (**A**) Schematic representation of *p21* genomic locus. p53 RE1, p53 response element 1; p53 RE2, p53 response element 2; TSS, transcription start site. (**B**) DNA damage induces p53 and RNF20 to the *p21* gene promoter region. HCT116 cells were treated with or without UV-C (20 J/m^2^). After 8 h recovery, ChIP analyses on the *p21* locus were performed using anti-p53 or anti-RNF20 antibodies. An irrelevant IgG was used for a control shown as the dotted lines. Quantitative PCR amplification regions are indicated in the schematic diagram. The sequence of each primer is shown in Supplementary Table S3. (**C**) ubH2B was enriched at the *p21* gene body region in response to DNA damage. ChIP analyses were performed with anti-ubH2B and anti-H2B antibodies. The relative enrichment of ubH2B was examined by the relative ratio of ubH2B vs. H2B. (**D**) p53 mediates the recruitment of RNF20 at the promoter region of *p21*. HCT116 cells were transfected with control siRNA (siNC), p53 siRNA, and RNF20 siRNA, respectively, following UV-C (20 J/m^2^). ChIP analyses on the *p21* locus were performed. The recruitment of RNF20 was examined by anti-RNF20 antibodies in ChIP assays. (**E**) Both p53 and RNF20 regulate ubH2B at the gene body region of *p21*. Knockdown of p53 or RNF20 was achieved by siRNA treatment. The relative ratio of ubH2B vs. H2B was examined by ChIP assays with anti-ubH2B and anti-H2B antibodies. (**F**) RNF20 is not required for the recruitment of p53. Following the siRNA treatment to knockdown p53 or RNF20, the enrichment of p53 was examined by ChIP assay with anti-p53 antibodies. (**G**) The CTD of p53 is required for the recruitment of RNF20. Full-length p53 and the D5 mutant were expressed in HCT116 p53^−/−^ cells. ChIP analyses on RNF20 were performed. Data are represented as mean ± standard deviation (SD) as indicated from three independent experiments. Significance of differences was evaluated by Student’s *t*-test. NS, nonsignificant; *P < 0.05; **P < 0.01; ***P < 0.001.

One major function of p53 is to induce gene transcription, such as *p21*, in response to DNA damage. As the RNF20/40 complex is also involved in transcriptional regulation, we examined the role of the RNF20/40 complex in the p53-dependent *p21* transcription. At the promoter region of *p21*, there are two previously mapped p53-binding sites (Zhang and Yu, 2011). With chromatin immunoprecipitation (ChIP) assays, we confirmed that p53 was enriched at the promoter regions of *p21* gene in HCT116 cells (Figure 3A and B). Moreover, following DNA damage induced by UV treatment, the levels of p53 at these regions were remarkably increased (Figure 3B). Consistently, we found that RNF20 was enriched together with p53 at the promoter region of *p21* gene. Moreover, as the RNF20/40 complex mediates H2B ubiquitination, we also examined the level of ubH2B. Notably, we only found a slight increase of ubH2B at the promoter region. However, the level of ubH2B was remarkably increased at the gene body region in response to DNA damage (Figure 3C). It is consistent with previous studies that ubH2B in the gene body region is associated with active gene transcription.

Next, since p53 interacts with the RNF20/40 complex, we asked whether p53 and the RNF20/40 complex regulate each other at the *p21* gene locus in response to UV treatment. We used siRNA to downregulate either p53 or RNF20 and found that loss of p53 remarkably reduced the level of the RNF20/40 complex at the promoter region of *p21* gene when cells were treated with UV (Figure 3D). Since the RNF20/40 complex mediates the level of ubH2B, loss of p53 also reduced the level of ubH2B at the gene body region of *p21* (Figure 3E). In contrast, lacking the RNF20/40 complex did not affect the localization of p53 at the promoter region of *p21*, although it abolished ubH2B (Figure 3F). Thus, these results suggest that p53 is an upstream regulator for the RNF20/40 complex during transcription regulation. We also expressed full-length p53 or the ΔCTD mutant (D5) that abolishes the interaction with the RNF20/40 complex (Supplementary Figure S4). Only the full-length p53 but not the ΔCTD mutant (D5) could restore the enrichment of the RNF20/40 complex at the promoter region of *p21* (Figure 3G). In addition, we also treated cells with MMS and obtained similar results in HCT116 cells (Supplementary Figure S5). Moreover, in p53^−/−^ HCT116 cells, we did not find the recruitment of RNF20/40 to the promoter region of *p21* for ubH2B (Supplementary Figure S6). Taken together, these results suggest that p53 mediates the recruitment of the RNF20/40 complex to the promoter region of *p21* and facilitates the RNF20/40 complex-dependent ubH2B at the gene body region of *p21*.

Moreover, to explore if it also occurs in other p53 target genes, we examined the recruitment of the RNF20/40 complex to *PUMA* locus. Similar to that at the *p21* locus, two p53-binding sites have been previously mapped at the promoter region of *PUMA* (Nakano and Vousden, 2001; Yu et al., 2001). Again, we observed that p53 mediated the recruitment of the RNF20/40 complex to *PUMA* locus for ubH2B (Supplementary Figure S7).

### RNF20/40 regulates *p21*-pre-mRNA maturation

**Figure 4:**
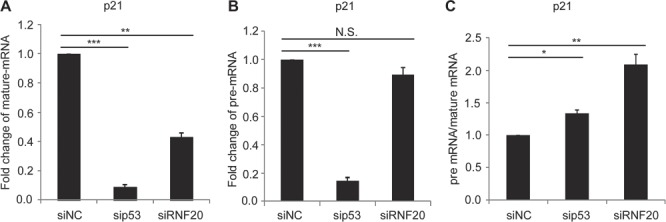
The RNF20/40 complex regulates *p21* pre-mRNA maturation. HCT116 cells were transfected with the indicated siRNA followed by UV-C (20 J/m^2^). After 8 h recovery, total RNA was extracted, and mature mRNA (**A**) and pre-mRNA (**B**) of *p21* were examined by quantitative PCR. (**C**) RNF20 plays a role in *p21* pre-mRNA processing. The fold changes of pre-mRNA vs. mature mRNA were compared. Lacking RNF20 induces relatively increased pre-mRNA of *p21*. The sequences of primers are shown in Supplementary Table S5. Data are represented as mean ± SD as indicated from three independent experiments. N.S., nonsignificant; *P < 0.05; **P < 0.01; ***P < 0.001.

It has been shown that both p53 and the RNF20/40 complex regulate *p21* gene transcription in response to DNA damage (Vogelstein and Kinzler, 1992; Kim et al., 2009). We further validated the results using quantitative reverse transcriptase-polymerase chain reaction (RT-PCR) and found that lacking p53 or the RNF20/40 complex significantly impaired the transcription of *p21* in HCT116 cells (Figure 4A). Since the RNF20/40 complex may be involved in transcription elongation, we also examined the pre-mRNA of *p21*. Surprisingly, we found that compared to the level of mature mRNA of *p21*, loss of the RNF20/40 complex only mildly affected the level of pre-mRNA of *p21*. In contrast, loss of p53 remarkably suppressed the transcription of pre-mRNA of *p21* (Figure 4B). Thus, these results suggest that p53 governs the transcription of pre-mRNA, whereas the RNF20/40 complex regulates not only the transcription but also the processing of the pre-mRNA into mature mRNA of *p21* (Figure 4C; Supplementary Figure S8A–C). Moreover, when cells were treated with MMS, we observed similar results (Supplementary Figure S8D–F), and only wild-type p53 but not the D5 mutant restored the ratio between pre-mRNA and mature mRNA of *p21* (Supplementary Figure S8G). We also examined and found that splicing of *PUMA* gene is also regulated by the RNF20/40 complex (Supplementary Figure S9). Taken together, these results suggest that p53 mediates the recruitment of the RNF20/40 complex for the splicing regulation of p53 target genes.

### PRPF8 recognizes ubH2B

**Figure 5:**
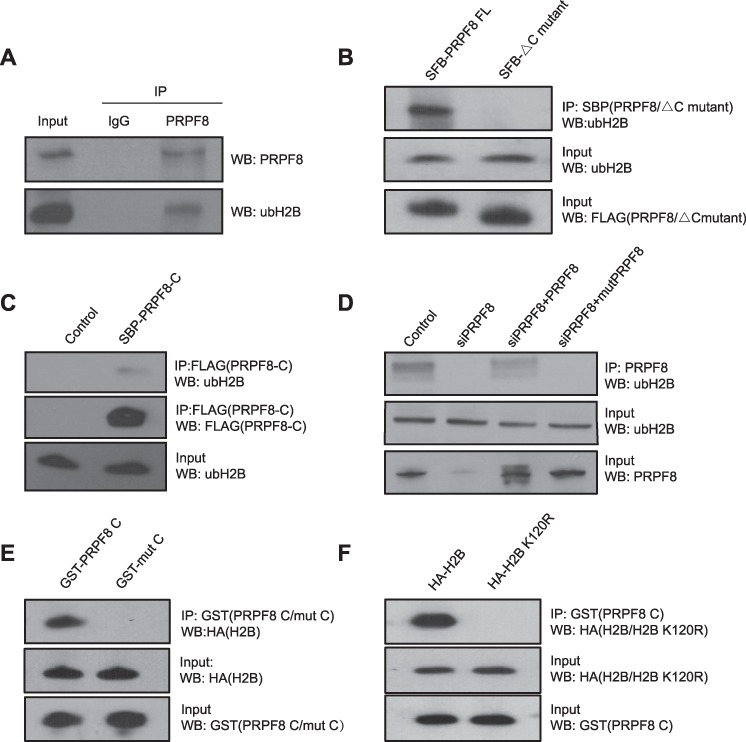
PRPF8 recognizes ubH2B via the C-terminal UBM. (**A**) RPRF8 recognizes ubH2B each other by co-IP and reciprocal co-IP assay. HCT116 cells were harvested. The chromatin fraction was isolated and the genomic DNA digested with benzonase to release nucleosomal histones. The lysates were examined by IP and western blot with indicated antibodies. (**B**) The deletion of C-terminal UBM of PRPF8 fails to recognize ubH2B. SFB-tagged full-length and UBM deletion mutant of PRPF8 (PRPF8 FL and PRPF8△C) were expressed in HCT116 cells. The interactions between PRPF8 FL or PRPF8△C and ubH2B were examined by IP and western blot. (**C**) The C-terminal UBM of PRPF8 recognizes ubH2B. SFB-tagged C-terminal UBM of PRPF8 (PRPF8-C) was expressed in HCT116 cells. The interaction between PRPF8-C and ubH2B was examined by IP and western blot. (**D**) The key residues in the UBM of PRPF8 are required for the interaction with ubH2B. Two key residues I2105 and L2106 in the UBM were mutated into alanines. This mutant (mutPRPF8) and wild-type PRPF8 were expressed in the PRPF8 knockdown HCT116 cells. The IP and western blot were performed with indicated antibodies. (**E**) Recombinant UBM of PRPF8 specifically pulls down ubH2B. GST-PRPF8 UBM or the I2105A/I2106A mutant was incubated with the histone fraction, followed by pull down with glutathione agarose and western blot analysis. (**F**) Ub null mutant of H2B cannot be recognized by UBM of PRPF8. K120 of H2B was mutated to Arg. HA-tagged H2B or the K120R mutant was expressed in HCT116 cells. The histone fraction was incubated with GST-PRPF8 UBM. Pull down and western blot assays were performed.

The processing of the pre-mRNA into mature mRNA is conducted by spliceosome, a large ribonucleoprotein complex. Thus, we hypothesize that the RNF20/40 complex may have functional interactions with spliceosome. Among these protein and RNA subunits in spliceosome, it has been reported that PRPF8 has a ubiquitin-binding motif (UBM) at the C-terminus (Bellare et al., 2006). As the RNF20/40 complex mediates ubH2B, we asked if PRPF8 recognized ubH2B. We first isolated the chromatin fraction and digested the genomic DNA with benzonase to release nucleosomal histones. With co-IP assays, we found that endogenous PRPF8 was associated with ubH2B (Figure 5A). Next, we generated the UBM deletion mutant of PRPF8 and found that the deletion mutant abolished the interaction (Figure 5B). Moreover, the UBM of PRPF8 itself was able to interact with ubH2B (Figure 5C). We mutated the key residues of UBM (I2105A and L2106A, named as mutPRPF8) and observed that the mutations abolished the interaction (Figure 5D). To further characterize the interaction, we generated the recombinant UBM of PRPF8 and found that it specifically pulled down ubH2B (Figure 5E) and the I2105A/L2106A mutant abolished the binding (Figure 5E). Finally, when we mutated K120 of H2B to Arg to abolish the ubH2B, the UBM of PRPF8 cannot recognize H2B anymore (Figure 5F). Taken together, these results suggest that the UBM of PRPF8 recognizes ubH2B.

### UbH2B is important for the recruitment of PRPF8 for *p21*

**Figure 6:**
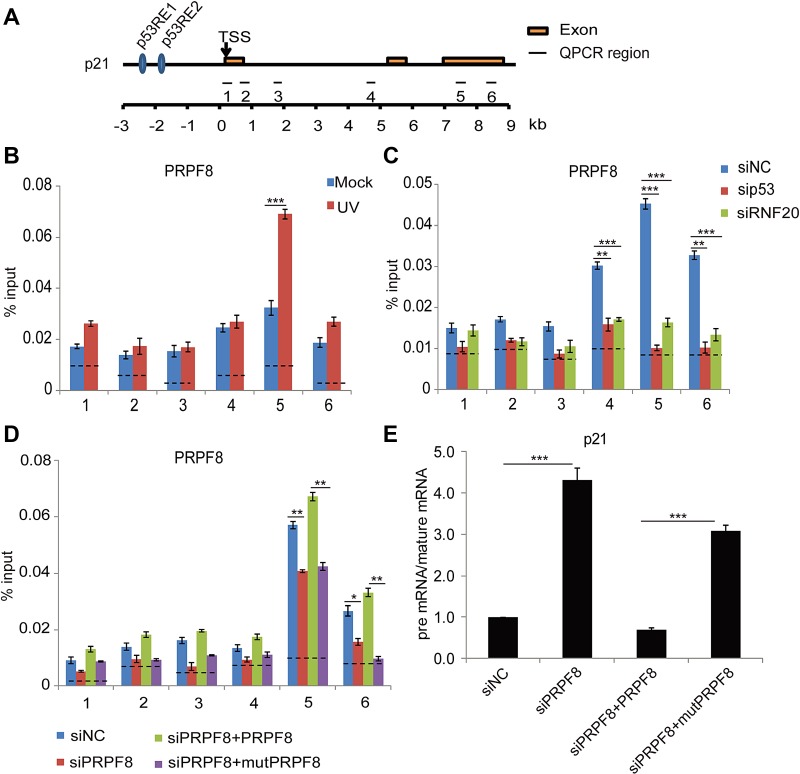
ubH2B mediates the recruitment of PRPF8 for pre-mRNA processing of *p21*. (**A**) Schematic representation of *p21* genomic locus. p53 RE1, p53 response element 1; p53 RE2, p53 response element 2; TSS, transcription start site. (**B**) PRPF8 is recruited to the gene body of *p21* upon DNA damage. HCT116 cells were treated with or without UV-C (20 J/m^2^). After 8 h recovery, ChIP analyses at the *p21* locus were performed using anti-PRPF8 antibody. An irrelevant IgG was used for negative control presented as the dotted lines. Primer pairs used for quantitative PCR following ChIP are indicated in schematic diagram. The primer sequences have been included in Supplementary Table S3. (**C**) Downregulation of p53 or the RNF20/40 complex by siRNA impairs the enrichment of PRPF8 at the gene body region of *p21*. HCT116 cells were transfected with the indicated siRNA followed by UV-C (20 J/m^2^). After 8 h recovery, ChIP analyses at the *p21* locus were performed using anti-PRPF8 antibody. (**D**) The C-terminal ub-binding mutant of PRPF8 (mutPRPF8) impairs the enrichment of PRPF8 at the gene body region of *p21*. Wild-type PRPF8 or the I2105A/I2106A mutant (mutPRPF8) was expressed in the siRNA knockdown cells. The ChIP assays were performed using anti-PRPF8 antibodies in response to UV-C (20 J/m^2^). (**E**) MutPRPF8 impairs the pre-mRNA processing of *p21*. HCT116 cells were treated with indicated siRNA. Full-length PRPF8 or the I2105A/I2106A mutant was re-introduced into the cells. The cells were irradiated with UV-C (20 J/m^2^). The relative changes of pre-mRNA vs. mature mRNA of *p21* were examined by quantitative PCR. The sequences of each primer are shown in Supplementary Table S5. Data are represented as mean ± SD as indicated from three independent experiments. *P < 0.05; **P < 0.01; ***P < 0.001.

**Figure 7:**
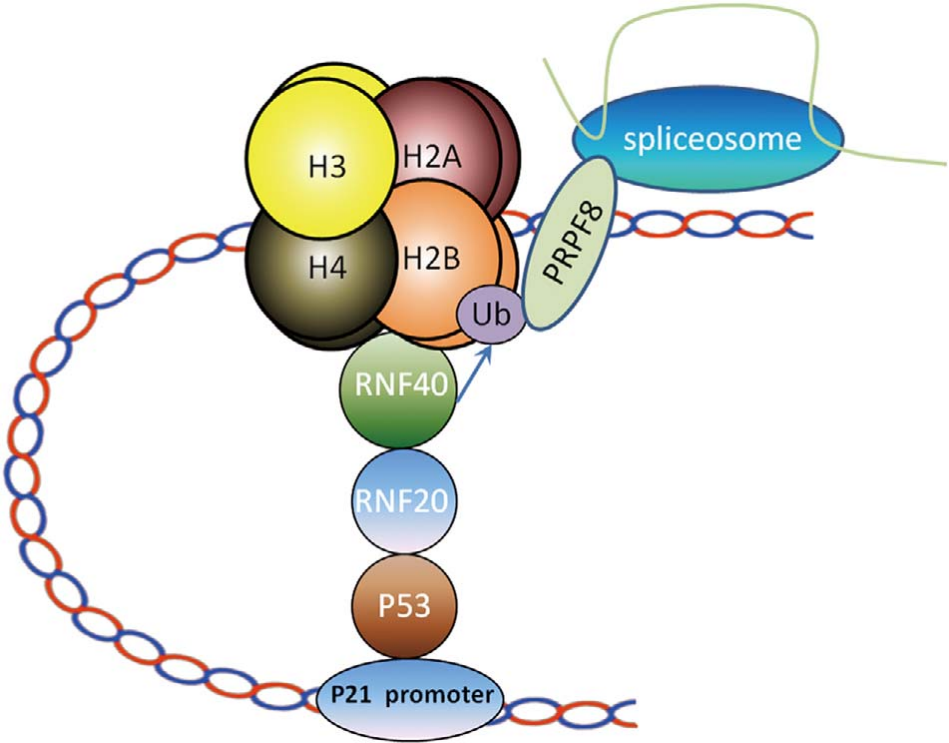
A model depicting the role of the RNF20/40 complex-dependent H2B ubiquitination in pre-mRNA processing. p53 recognizes *p21* promoter and recruits RNF20/40 to gene transcription sites for H2B ubiquitination. PRPF8, a subunit of spliceosome, recognizes ubH2B via the C-terminal UBM. UbH2B mediates the recruitment of PRPF8 for pre-mRNA processing of *p21*.

As a subunit in spliceosome, PRPF8 is involved in the mRNA splicing. Similar to ubH2B, PRPF8 was enriched in the gene body region of *p21* and *PUMA* loci following DNA damage (Figure 6A and B; Supplementary Figure S10A). Notably, when we knocked down p53 and the RNF20/40 complex by siRNA to downregulate ubH2B, the enrichment of PRPF8 at the gene body regions was impaired (Figure 6C; Supplementary Figure S10B). Moreover, only wild-type PRPF8 but not the mutPRPF8 was recruited to the gene body regions (Figure 6D; Supplementary Figure S10C), suggesting that the RNF20/40 complex-dependent ubH2B mediates the recruitment of PRPF8 to the gene body region. Consistently, only in the presence of wild-type PRPF8, the pre-mRNA of *p21* and *PUMA* was processed into mature mRNA. However, in the presence of mutPRPF8 that loses the interaction with ubH2B, pre-mRNA processing by spliceosome was impaired (Figure 6E; Supplementary Figure S10D and E). We also validated the results in cells treated with MMS (Supplementary Figure S11). In particular, the recruitment of PRPF8 was suppressed in p53^−/−^ HCT116 cells (Supplementary Figure S11B). Moreover, we examined another spliceosome subunit DHX8, which is associated with PRPF8 as well as mutPRPF8. Loss of DHX8 also impaired normal splicing of *p21* (Supplementary Figure S12). Taken together, these results show that ubH2B mediates the recruitment of PRPF8 for pre-mRNA processing, which is one of key functions of the RNF20/40 complex in transcription regulation (Figure 7).

## Discussion

In this study, we have demonstrated that the RNF20/40 complex is a functional partner of p53 in the gene transcription. We have mapped the interaction region. Notably, the CTD of p53 mediates the interaction with the RNF20/40 complex. It has been shown that the CTD of p53 plays an important role in transcription elongation (Beckerman and Prives, 2010). However, the underlying mechanism remains elusive. Here, our results demonstrate the CTD recruits the RNF20/40 complex to *p21* and *PUMA* gene promoter regions. It has been shown that the CTD may also interact with genomic DNA (Liu and Kulesz-Martin, 2006). Currently, it is unclear if genomic DNA plays any role for interaction between p53 and RNF20/40 complex. However, in our protein affinity purification, we do not expect that genomic DNA is included in the protein complex as we did not use any nuclease to release chromatin-binding complex. Thus, it is likely that the interaction between p53 and the RNF20/40 complex is DNA-independent. Moreover, it has been shown that several lysine residues in the CTD undergo posttranslational modifications, especially acetylation, during transcription regulation (Avantaggiati et al., 1997; Gu and Roeder, 1997; Sakaguchi et al., 1998; Liu and Bodmer, 2006). Here, we do not know if these modifications affect the recruitment of the RNF20/40 complex. Future in-depth analysis on these modifications as well as detailed structural analysis will reveal the binding mode between p53 and the RNF20/40 complex. Nevertheless, our studies reveal a functional partner of p53 in the regulation of transcription elongation.

Interestingly, ubH2B at the promoter region is not increased. Instead, it is the level of ubH2B at gene body region that is increased. It is possible that the RNF20/40 complex is recruited to the promoter region by p53. Along with the transcription elongation, the RNF20/40 complex slides into the gene body region for ubH2B, where the RNF20/40 complex does not tightly associate with the chromatin. Thus, with high-salt extraction, we found that the RNF20/40 complex was mainly enriched at the promoter region. Alternatively, the RNF20/40 complex localizes to the promoter region and mediates ubH2B at the gene body region through the high-order loop structure of the chromatin.

Moreover, ubH2B regulate not only transcription elongation but also mRNA splicing. It has been shown that p53 modulates mRNA splicing in response to genotoxic stress (Balakrishnan and Gross, 2008). However, p53 mediates other earlier steps of transcription such as transcription initiation. And loss of p53 abolishes the targeting gene transcription. Thus, it is difficult to examine the role of p53 in mRNA splicing, the following step after transcription. Here, we have shown that p53 mediates the recruitment of the RNF20/40 complex, which in turn regulates the mRNA splicing of *p21* and *PUMA*. Moreover, we have revealed that the UBM of PRPF8 recognizes ubH2B at the gene bodies of *p21* and *PUMA*. It is likely that this interaction mediates the recruitment of the spliceosome for the mRNA splicing of *p21* and *PUMA*. Thus, this p53-RNF20/40-ubH2B- PRPF8 pathway plays a key role for p53-dependent mRNA splicing in response to genotoxic stress. Notably, loss of ubH2B also suppresses transcription elongation, which may indirectly affect mRNA splicing. It is possible that multiple pathways act together to regulate mRNA splicing, and ubH2B-mediated splicing machinery is only one of these pathways. Future comprehensive analysis of mRNA splicing will reveal the detailed underlying mechanisms.

The structure of the spliceosome has been solved recently (Shi, 2017). Interestingly, a large portion of PRPF8, a subunit in the spliceosome, extends toward the outside of the complex, especially the UBM (Shi, 2017). It indicates that this UBM facilitates the interaction with its functional partners. Here, we have shown that this UBM recognizes ubH2B. However, more detailed binding analysis in future may reveal the recognition of the specific ubiquitination site on nucleosomal histone.

In addition to the RNF20/40 complex, p53 has many other functional partners. Due to the specific purification condition, we only found that p53 associated with the RNF20/40 complex on the chromatin under the relatively high-salt condition. It is possible that the RNF20/40 complex functions together with other p53 partners for other steps during gene transcription. Moreover, our study focuses on two well-known p53 target genes. It would be important for exploring other p53 targets to validate the general mechanism of p53-dependent splicing regulation.

## Materials and methods

### Plasmids, antibodies, and other materials

Human full-length RNF20, RNF40, p53, and PRPF8 were cloned into the SFB vector (S-FLAG-SBP-tagged). Human p53 was cloned into pCMV-HA vector. RNF20 D1–D8, RNF40 D1–D14, and p53D1–D5 mutants were cloned into the pCMV-HA vector. Human PRPF8 and its mutant (I2105A, L2106A, and △C mutant) were cloned into the pEGFP-N1 vector or SFB.

Polyclonal anti-human PRPF8, anti-RNF20, anti-RNF40, and anti-DHX8 antibodies were purchased from Bethyl. Monoclonal anti-human p53 was purchased from Santa Cruz. Anti-HA, anti-FLAG, anti-GST, and anti-β-actin antibodies were purchased from Sigma. Anti-monoubiquitinated H2B antibody and anti-H2B antibody were purchased from GeneTex. Nonimmune mouse IgG (Sigma) was used as a negative control.

Cells were maintained in Dulbecco's Modified Eagle Medium (DMEM) with 10% fetal serum and cultivated at 37 C in 5% CO_2_ (*v*/*v*). All cell lines were purchase form American Type Culture Collection.

### RNA interference

siRNAs were synthesized by Thermo Scientific Dharmacon. The siRNA sequences targeting RNF20, p53, and PRPF8 are as follows: GAAGGCAGCUGUUGAAGAUdTdT (RNF20), GACAGAUCUUCUUCAGGAAdTdT (RNF20–2), CACUACAACUACAUGUGUAdTdT (p53), CTACUUCCUGAAAACAACGdTdT (p53–2), CCUGUAUGCCUGACCGUUUdTdT (PRPF8), and GGACUGCACUAGCCCUGAAdTdT (DHX8). siRNAs were transfected into cells using Oligofectamine 2000 (Invitrogen) according to manufacturer’s instructions.

### Protein purification and size-exclusion chromatography

Purification of SFB triple-tagged protein (S, FLAG, and SBP tags) was described previously (Kim et al., 2007). To search for binding partners of p53, we harvested HCT116 and K562 cells stably expressing SFB-p53, respectively, and washed them with phosphate buffered saline (PBS). Cells were lyzed with 30 ml ice-cold NETN100 buffer. The pellets were incubated with NETN300 buffer. The soluble fraction was incubated with 0.5 ml streptavidin-conjugated agarose beads. The beads were washed with NETN buffer three times. Associated proteins were eluted with 2 mM biotin in PBS and further incubated with 0.05 ml S beads (Novagen). The bound proteins were eluted with sodium dodecyl sulphate (SDS) sample loading buffer and analyzed with 10% SDS-polyacrylamide gel electrophoresis (SDS-PAGE) and MS. Cells expressing empty vector were used as purification controls.

### ChIP assay

ChIP assay was performed according to the protocol described by Upstate. Briefly, cells were fixed in 1% formaldehyde for 10 min at room temperature before quenching with 125 mM glycine. Cells were washed twice with ice-cold 1× PBS buffer. Cell pellets were lyzed in 1 ml of SDS buffer (1% SDS, 10 mM EDTA, 50 mM Tris–HCl (pH 8.0), containing protease inhibitors) for 10 min on ice and sonicated lysate to shear DNA to an average fragment size of 500–1000 bp. Chromatin extracts were cleared by centrifugation for 10 min and diluted 1:10 in a buffer containing 20 mM Tris–HCl (pH 8.0), 150 NaCl, 2 mM EDTA, and 1% Triton X-100 to reduce SDS concentration. Antibody was used with 50 μl protein A/G agarose plus salmon sperm DNA (Millipore) to preclear the diluted chromatin for 1 h. Then 50 μl of each sample was collected to serve as input and store at −20 ˚C until further use. The cleared supernatant was incubated with 5 μg antibody or control IgG overnight. Agarose beads were used to precipitate immune complexes for 2 h before washing in buffers of increasing stringency as follows: once in 20 mM Tris–HCl (pH 8.0), 150 mM NaCl, 2 mM EDTA, 1% SDS, and 1% Triton X-100; once in 20 mM Tris–HCl (pH 8.0), 500 mM NaCl, 2 mM EDTA, 1% SDS, and 1% Triton X-100; once in 10 mM Tris–HCl (pH 8.0), 1 mM EDTA, 1% deoxycholic acid, 1% NP-40, and 0.25 M LiCl; and twice with Tris-EDTA buffer. Beads were centrifuged at 2000× *g* for 1 min to pellet. DNA was eluted from beads in 100 mM NaHCO3 and 1% SDS at 65°C overnight followed by RNase A and protease K treatment. DNA was recovered by phenol–chloroform–ethanol purification. Primers for ChIP quantitative PCR are summarized in Supplementary Tables S3 and S4. The mean value was calculated by three independent experiments.

### Cell lysis, immunoprecipitation, and western blotting

Cells were lyzed with NETN100 buffer. Immunoprecipitation and western blotting were performed following standard protocol as described previously (Kim et al., 2007). For analysis interaction of p53 and chromatin under different lysis conditions, HCT116 cells were lyzed with NETN buffer containing 100 and 300 mM NaCl, respectively. Samples were used for further western blot analysis.

### Quantitative RT-PCR assay

Total RNA was prepared using Trizol (Gibco-Invitrogen) and used for synthesis of first strand cDNA with Superscript II reverse transcriptase (Invitrogen). DNase I was used to remove all genomic DNA. Quantitative PCR was performed using Power SYBR green PCR master mix in 7300 Real-Time PCR System (Applied Biosystems). Primers for quantitative PCR reactions are summarized in Supplementary Tables S5 and S6. *GAPDH* mRNA was used for normalization. The mean value was calculated from three independent experiments.

## Supplementary Material

Supplemental_Information-20190425_mjz045
