# Supplementary material for: The RNF20/40 complex regulates p53-dependent gene transcription and mRNA splicing
Source: J Mol Cell Biol. 2019 Jun 1;12(2):113–24. doi: 10.1093/jmcb/mjz045 (PMC7109600; doi:10.1093/jmcb/mjz045)
Supplement: Supplemental_Information-20190425_mjz045 [file supplemental_information-20190425_mjz045.pdf]

Supplementary Material

The RNF20/40 complex regulates p53-dependent gene transcription and mRNA splicing

Chen Wu[1, 2], Yaqi Cui[1, 2], Xiuhua Liu[1], Feng Zhang[3], Lin-Yu Lu[4, 5], and Xiaochun Yu[2, *]

[1]College of Life Sciences, Hebei University, Baoding, 071000, Hebei, PR China

[2]Department of Cancer Genetics and Epigenetics, Beckman Research Institute, City of Hope, 1500 E. Duarte Rd, Duarte, California, 91010, USA

[3]College of Life and Environment Sciences, Shanghai Normal University, Shanghai, China

[4]Key Laboratory of Reproductive Genetics, Ministry of Education and Women's Reproductive Health Laboratory of Zhejiang Province, Women's Hospital, School of Medicine, Zhejiang University, Hangzhou, Zhejiang, China.

[5]Institute of Translational Medicine, Zhejiang University, Hangzhou, Zhejiang, China.

Correspondence should be addressed to: Xiaochun Yu (xyu@coh.org)

Content:

I. Supplementary Figures 4

Supplementary Figure S1. p53 was expressed and tightly associated with genomic DNA in response to DNA damage. 4

Supplementary Figure S2. Exogenous and endogenous p53 expression in HCT116 and K562 cells. 5

Supplementary Figure S3. p53 interacts with RNF20 and RNF40...6

Supplementary Figure S4. Wild type p53 or the D5 mutant was expressed in HCT116 p53 -/- cells. 7

Supplementary Figure S5. p53 mediates the recruitment of the RNF20/40 complex to the promoter region at *p21* loci in response to MMS treatment. 8

Supplementary Figure S6. p53 mediates the recruitment of the RNF20/40 complex to the promoter region of *p21*. 10

Supplementary Figure S7. p53 mediates the recruitment of the RNF20/40 complex to the promoter region at *PUMA* loci in HCT116 cells. 12

Supplementary Figure S8. The RNF20/40 complex regulates *p21* pre-mRNA maturation in response to DNA damage. 14

Supplementary Figure S9. The RNF20/40 complex regulates *PUMA* pre-mRNA maturation. 16

Supplementary Figure S10. ubH2B mediates the recruitment of PRPF8 for the pre-mRNA processing of *PUMA* in HCT116 cells...17

Supplementary Figure S11. PRPF8 impairs the pre-mRNA processing of *p21* in HCT116 cells. 19

Supplementary Figure S12. DHX8 impairs the pre-mRNA processing of *p21* in HCT116 cells. 21

II. Supplementary tables..23

Supplementary Table S1. Affinaty purification and mass spectrometry analysis of p53 in HCT116 cell..23

Supplementary Table S2. Affinaty purification and mass spectrometry analysis of p53 in K562 cell..30

Supplementary Table S3. Primer sequences for ChIP-qPCR analysis of the *p21* locus........34

Supplementary Table S4. Primer sequences for ChIP-qPCR analysis of the *PUMA* locus. .34

Supplementary Table S5. Primer sequences for RT-PCR analysis of *p21* mature and pre-mRNA expression. 35

Supplementary Table S6. Primer sequences for RT-PCR analysis of *PUMA* mature and pre-mRNA expression. 35

I. Supplementary Figures

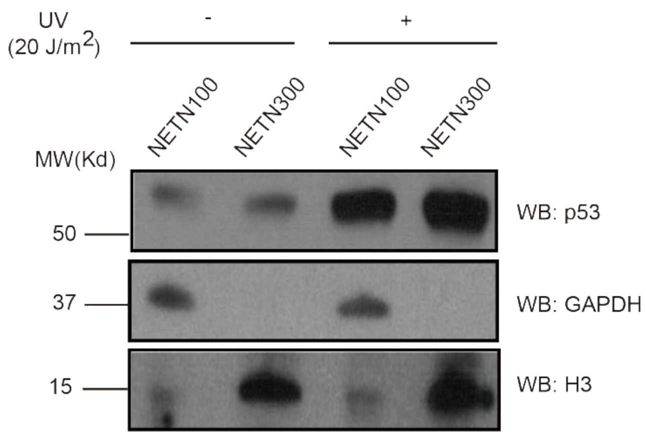

Supplementary Figure S1. p53 was expressed and tightly associated with genomic DNA in response to DNA damage.

HCT116 cells were treated with or without UV-C (20 J/m2). After 8h, cells were lysed by NETN100 (lysis buffer with 100 mM NaCl) to obtain the soluble fraction. The pellets were further treated with NETN300 (lysis buffer with 300 mM NaCl) and 250 Units benzonase to extract the chromatin fraction. Both fractions were examined by Western blotting. GAPDH and histone H3 were used as a loading control for each fraction.

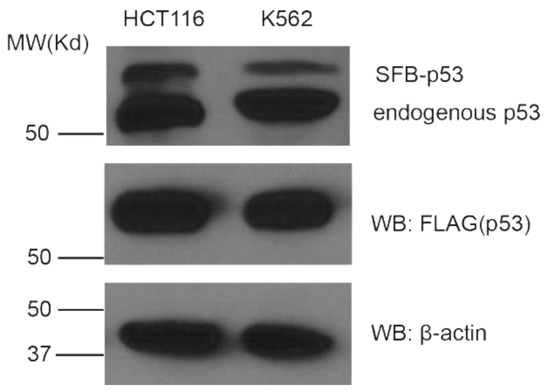

Supplementary Figure S2. Exogenous and endogenous p53 expression in HCT116 and K562 cells.

HCT116 and K562 cells were transfected stably with SFB-p53 plasmid. Following UV-C (20 J/m2), the expression levels of exogenous p53 and endogenous wild-type p53 in both K562 and HCT116 were determined by Western blot with anti-p53 antibody. Cells were lysed in NETN300 buffer. β-actin was used as the protein loading control.

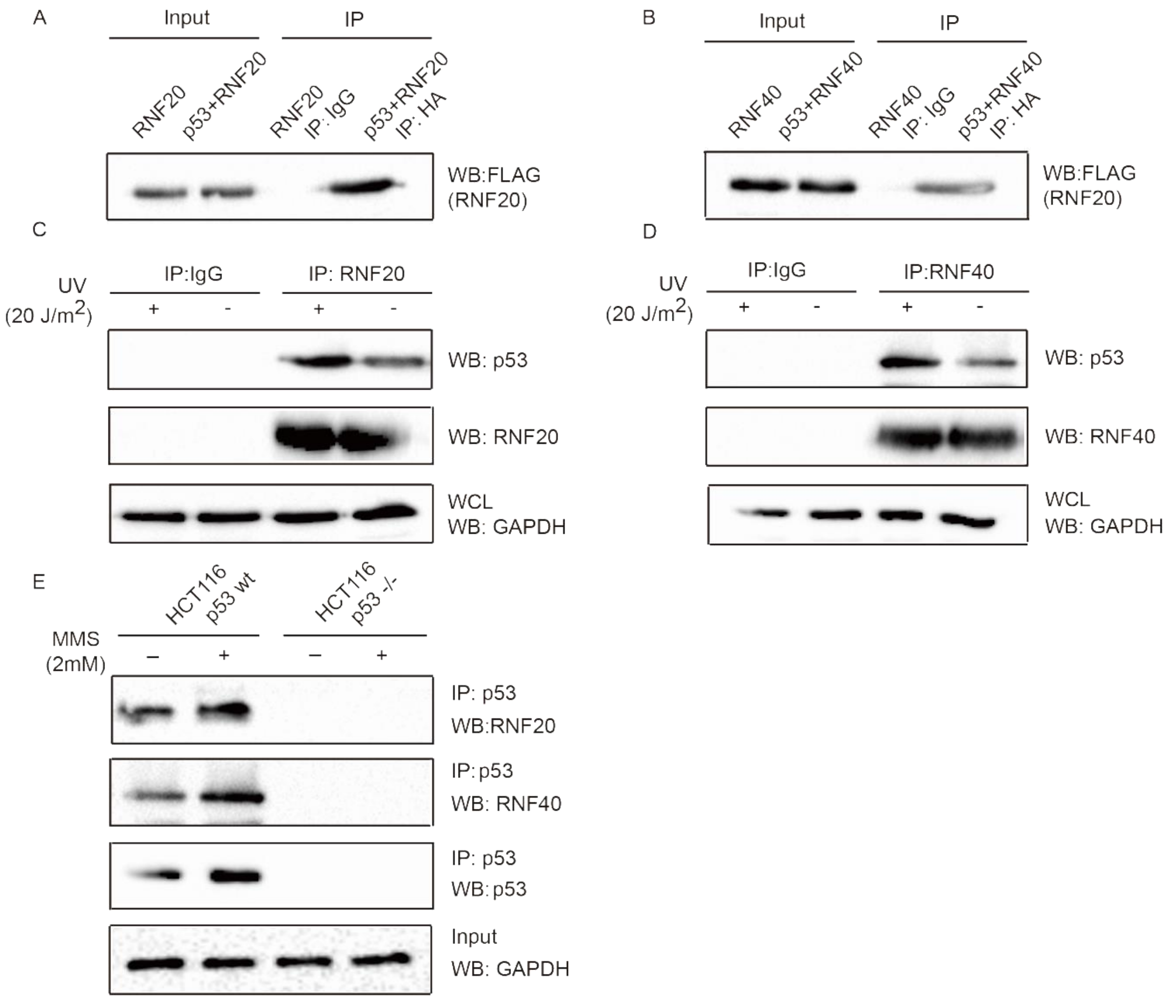

Supplementary Figure S3. p53 interacts with RNF20 and RNF40.

HCT116 cells were transfected with HA-p53 and SFB-RNF20 or SFB-RNF20 (A) or HA-p53 and SFB-RNF40 or SFB-RNF40 (B). Cells were lyzed with NETN300 buffer. Cell extracts were examined by IP and Western blot with indicated antibodies using unspecific IgG as a negative control. (C and D) p53 associates with endogenous RNF20/40. HCT116 cells were treated with UV-C (20 J/m2). After 8 hours recovery, cells were lyzed with NETN300 buffer, and lysates were examined by IP and Western blot with indicated antibodies. GAPDH was used as the protein loading controls. (E) p53 -/- HCT116 cells were used to as a negative control to confirm the specificity of IP of p53 protein.

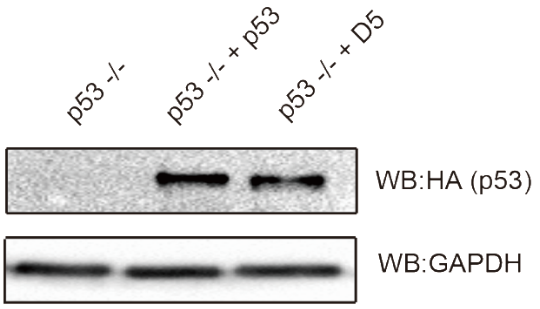

Supplementary Figure S4. Wild type p53 or the D5 mutant was expressed in HCT116 p53 -/- cells.

The expression was examined by Western blot.

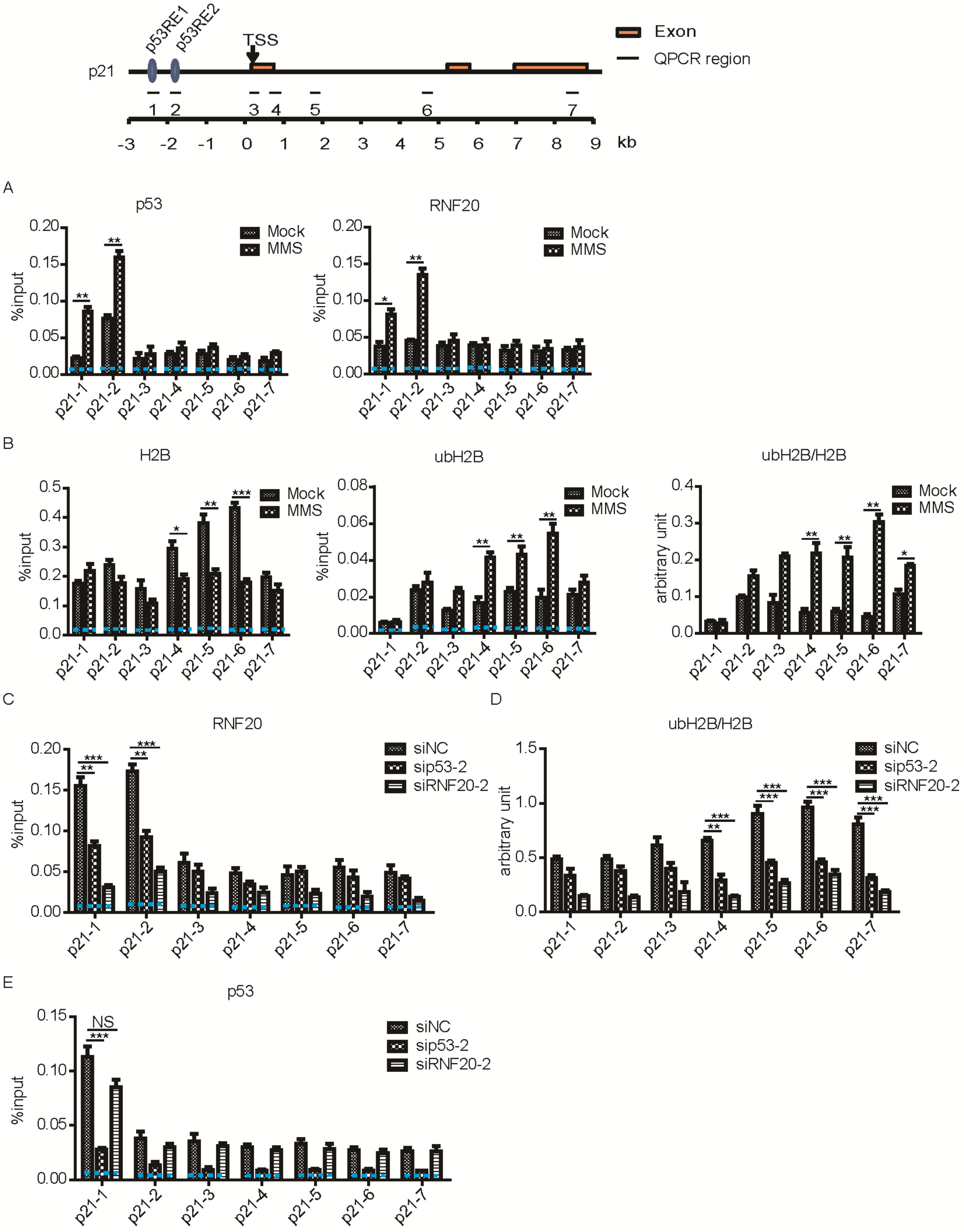

Supplementary Figure S5. p53 mediates the recruitment of the RNF20/40 complex to the promoter region at *p21* loci in response to MMS treatment.

(A) DNA damage induces p53 and RNF20 to the *p21* gene promoter region. HCT116 cells were treated with or without 2mM MMS. After one hour, ChIP analyses on the *p21* locus were performed using anti-p53 and anti-RNF20 antibodies. An irrelevant IgG was used for a control shown as the dotted lines. Primers of *p21* used for q-PCR are included in Table S3. (B) ubH2B was enriched at the *p21* gene body region in response to MMS. ChIP analyses of H2B and ubH2B/H2B were included. (C) p53 mediates the recruitment of RNF20 at the promoter region of *p21*. HCT116 cells were transfected with another control siRNA (siNC), p53 siRNA and RNF20 siRNA respectively. ChIP analyses on the *p21* locus were performed. (D) Both p53 and RNF20 regulate ubH2B at the gene body region of *p21*. (E) RNF20 is not required for the recruitment of p53. Data are represented as mean ± SD as indicated from three independent experiments. N.S.: Non Significant; *: Statistically Significant ($p < 0.05$); **: Statistically Significant ($p < 0.01$); ***: Statistically Significant ($p < 0.001$).

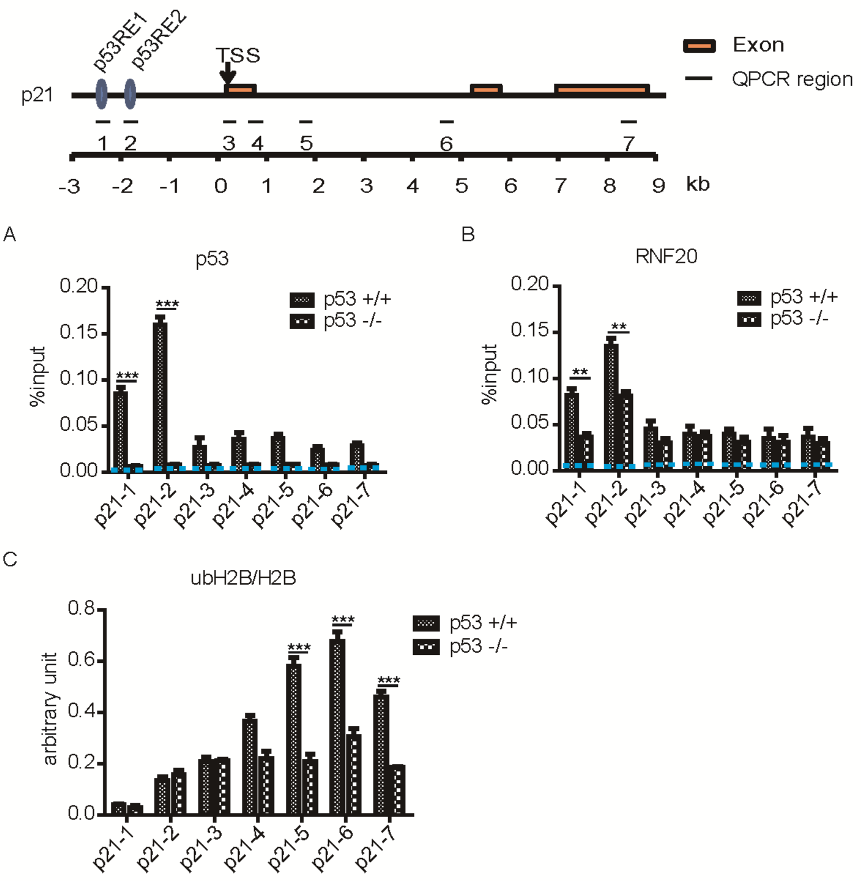

Supplementary Figure S6. p53 mediates the recruitment of the RNF20/40 complex to the promoter region of *p21*.

p53 -/- HCT116 cells were performed by ChIP assay as a negative control for p53 +/+ HCT116. DNA damage induces p53 and RNF20 to the *p21* gene promoter region.HCT116 cells were treated with or without 2mM MMS. After one hour, ChIP analyses on the *p21* locus were performed using anti-p53 (A) or anti-RNF20 (B) antibodies. An irrelevant IgG was used for a negative control shown as the dotted lines. Q-PCR amplification regions are indicated in the schematic diagram. The sequences of each primer are shown in Supplemental Table S3. (C) ubH2B was enriched at the *p21* gene body region in response to DNA damage. ChIP analyses were performed with anti-ubH2B and anti-H2B antibodies. The relative enrichment of ubH2B was examined by the relative ratio of ubH2B vs H2B. Data are represented as mean ± SD as

indicated from three independent experiments. Significance of differences was evaluated by Student's t test. N.S.: Non-Significant; *: Statistically Significant ($p < 0.05$); **: Statistically Significant ($p < 0.01$); ***: Statistically Significant ($p < 0.001$).

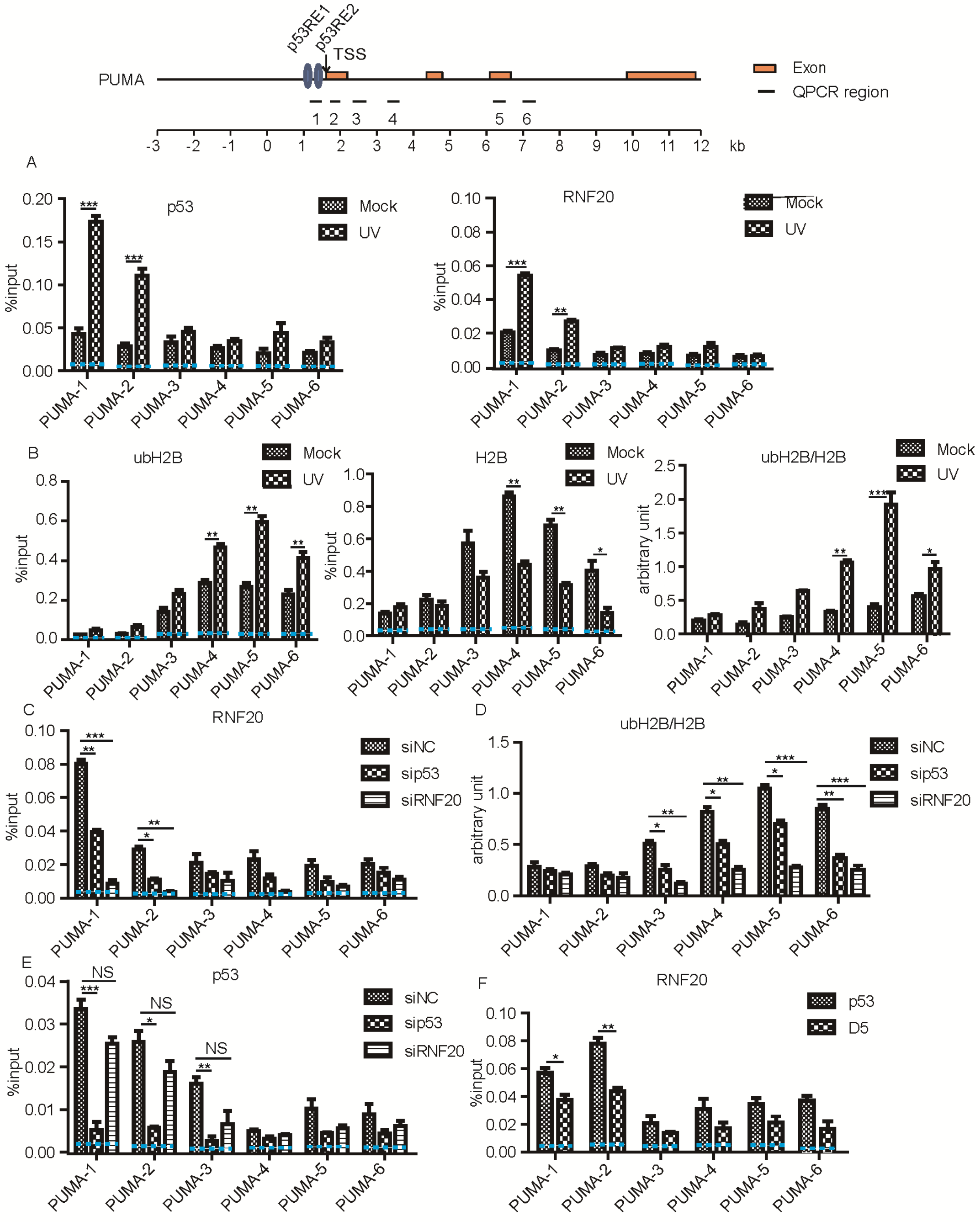

Supplementary Figure S7. p53 mediates the recruitment of the RNF20/40 complex to the promoter region at *PUMA* loci in HCT116 cells.

(A) DNA damage induces p53 and RNF20 to the *PUMA* gene promoter region. HCT116 cells were treated with or without UV-C (20 J/m2). After 8 hours recovery, ChIP analyses on the *PUMA* locus were performed using anti-p53 and anti-RNF20 antibodies. An irrelevant IgG was used for a control shown as the dotted lines. Primers of *PUMA* used for q-PCR are included in Table S4. (B) ubH2B was enriched at the *PUMA* gene body region in response to DNA damage. ChIP analyses of H2B and ubH2B/H2B were included. (C) P53 mediates the recruitment of RNF20 at the promoter region of *PUMA*. HCT116 cells were transfected with control siRNA (siNC), p53 siRNA and RNF20 siRNA respectively. ChIP analyses on the *PUMA* locus were performed. (D) Both p53 and RNF20 regulate ubH2B at the gene body region of *PUMA*. (E) RNF20 is not required for the recruitment of p53. (F) The CTD of p53 is required for the recruitment of RNF20. Full length p53 and the D5 mutant were expressed in HCT116 p53 -/- cells. ChIP analyses on RNF20 were performed. Data are represented as mean ± SD as indicated from three independent experiments. N.S.: Non Significant; *: Statistically Significant ($p < 0.05$); **: Statistically Significant ($p < 0.01$); ***: Statistically Significant ($p < 0.001$).

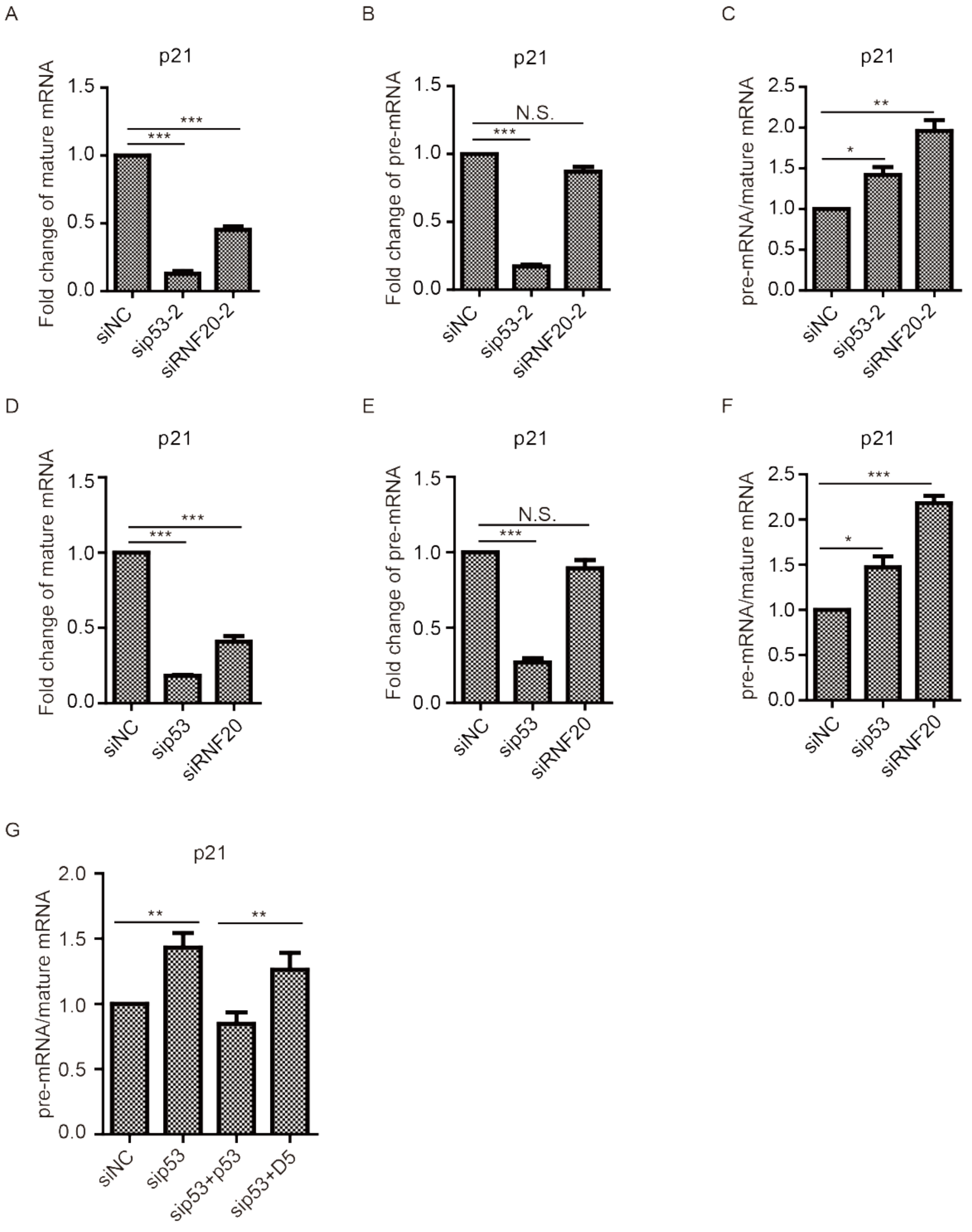

Supplementary Figure S8. The RNF20/40 complex regulates *p21* pre-mRNA maturation in response to DNA damage.

HCT116 cells were transfected with the indicated another siRNA followed by UV-C (20 J/m^2). After 8 hours, mature mRNA(A) and pre-mRNA(B) of *p21* were examined by q-PCR. (C) RNF20 plays an important role in *p21* pre-mRNA processing in response to UV treatment. The fold changes of pre-mRNA vs. mature mRNA were compared. Lacking RNF20 induces

relatively increased pre-mRNA of *p21* in HCT116 cells. We did the same assay followed by 2mM MMS in replacement of UV treatment. After 1 hours, mature mRNA(C) and pre-mRNA. (D) of *p21* were examined by q-PCR. (E) RNF20 plays an important role in *p21* pre-mRNA and mature mRNA(F) processing in response to MMS treatment. (G) The CTD mutation (D5) of p53 affects mRNA splicing of *p21*. The primer sequences for q-PCR at *p21* loci have been included in Supplemental Table S5. Data are represented as mean ± SD as indicated from three independent experiments. N.S.: Non-Significant; *: Statistically Significant ($p < 0.05$); **: Statistically Significant ($p < 0.01$); ***: Statistically Significant ($p < 0.001$).

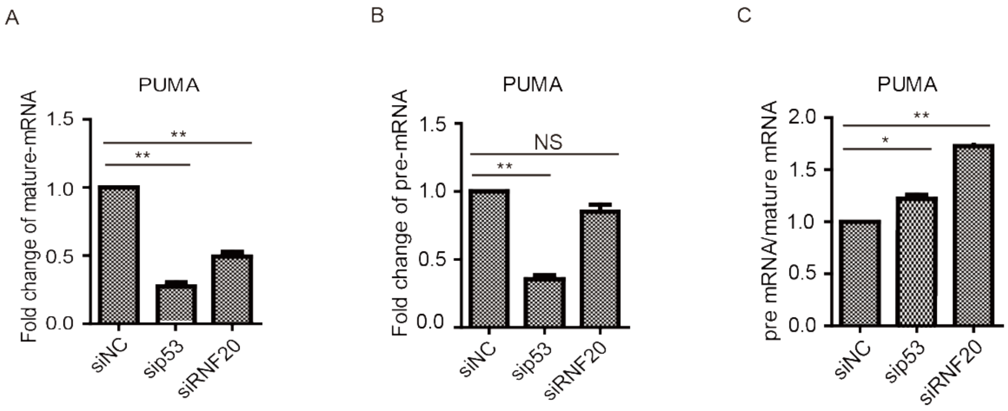

Supplementary Figure S9. The RNF20/40 complex regulates *PUMA* pre-mRNA maturation.

HCT116 cells were transfected with the indicated siRNA followed by UV-C (20 J/m^2). After 8 hours recovery, mature mRNA (A) and pre-mRNA (B) of *PUMA* were examined by q-PCR. (C) RNF20 plays an important role in *PUMA* pre-mRNA processing in HCT116 cells. The fold changes of pre-mRNA vs. mature mRNA were compared. Lacking RNF20 induces relatively increased pre-mRNA of *PUMA* in HCT116 cells. The primer sequences for q-PCR at *PUMA* loci have been included in Supplemental Table S6. Data are represented as mean ± SD as indicated from three independent experiments. *N.S.*: Non-Significant; *: Statistically Significant ($p < 0.05$); **: Statistically Significant ($p < 0.01$); ***: Statistically Significant ($p < 0.001$).

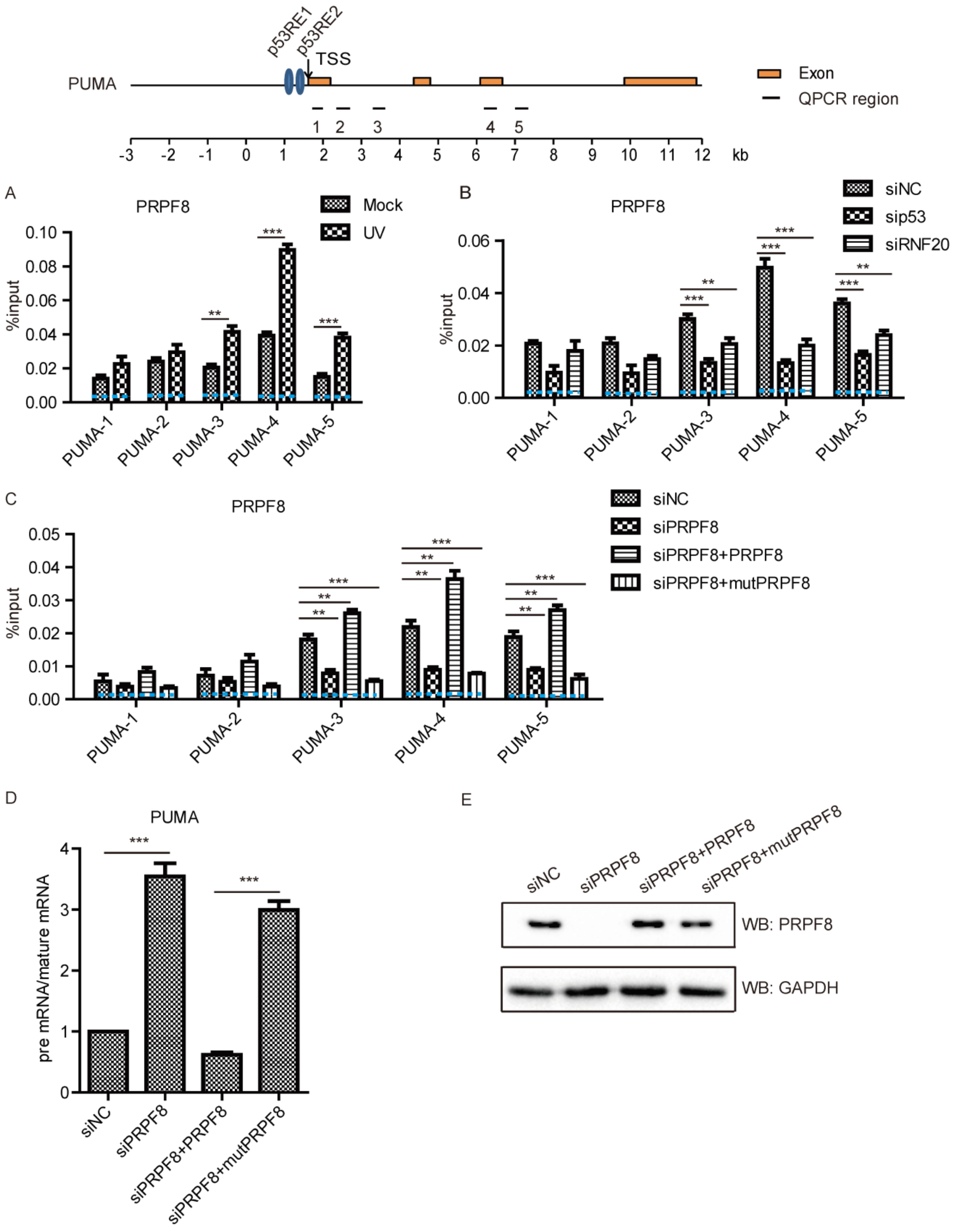

Supplementary Figure S10. ubH2B mediates the recruitment of PRPF8 for the pre-mRNA processing of *PUMA* in HCT116 cells.

(A) PRPF8 is recruited to the gene body of *PUMA* upon DNA damage. HCT116 cells were treated with or without UV-C (20J/m^2). ChIP analyses at the *PUMA* locus were performed using anti-PRPF8 antibodies. An irrelevant IgG was used for negative control presented as the dotted lines. Primers used for q-PCR are included in Supplemental Table S4. (B) Downregulation of p53 or the RNF20/40 complex by siRNA impairs the enrichment of PRPF8 at the gene body region of *PUMA* in HCT116 cells. (C) The I2105A/I2106A mutant of PRPF8 (mutPRPF8) impairs the enrichment of PRPF8 at the gene body region of *PUMA*. (D) MutPRPF8 impairs the pre-mRNA processing of *PUMA*. HCT116 cells were treated with indicated siRNA. Full length PRPF8 or mutPRPF8 were re-introduced into the cells. The cells were treated with UV-C (20 J/m^2). The relative changes of pre-mRNA vs. mature mRNA of *PUMA* were examined by q-PCR. The primer sequences have been included in Supplemental Table S6. (E) The expressions of PRPF8 and mutant PRPF8 were examined by western blot. Data are represented as mean ± SD as indicated from three independent experiments. *: Statistically Significant ($p < 0.05$); **: Statistically Significant ($p < 0.01$); ***: Statistically Significant ($p < 0.001$).

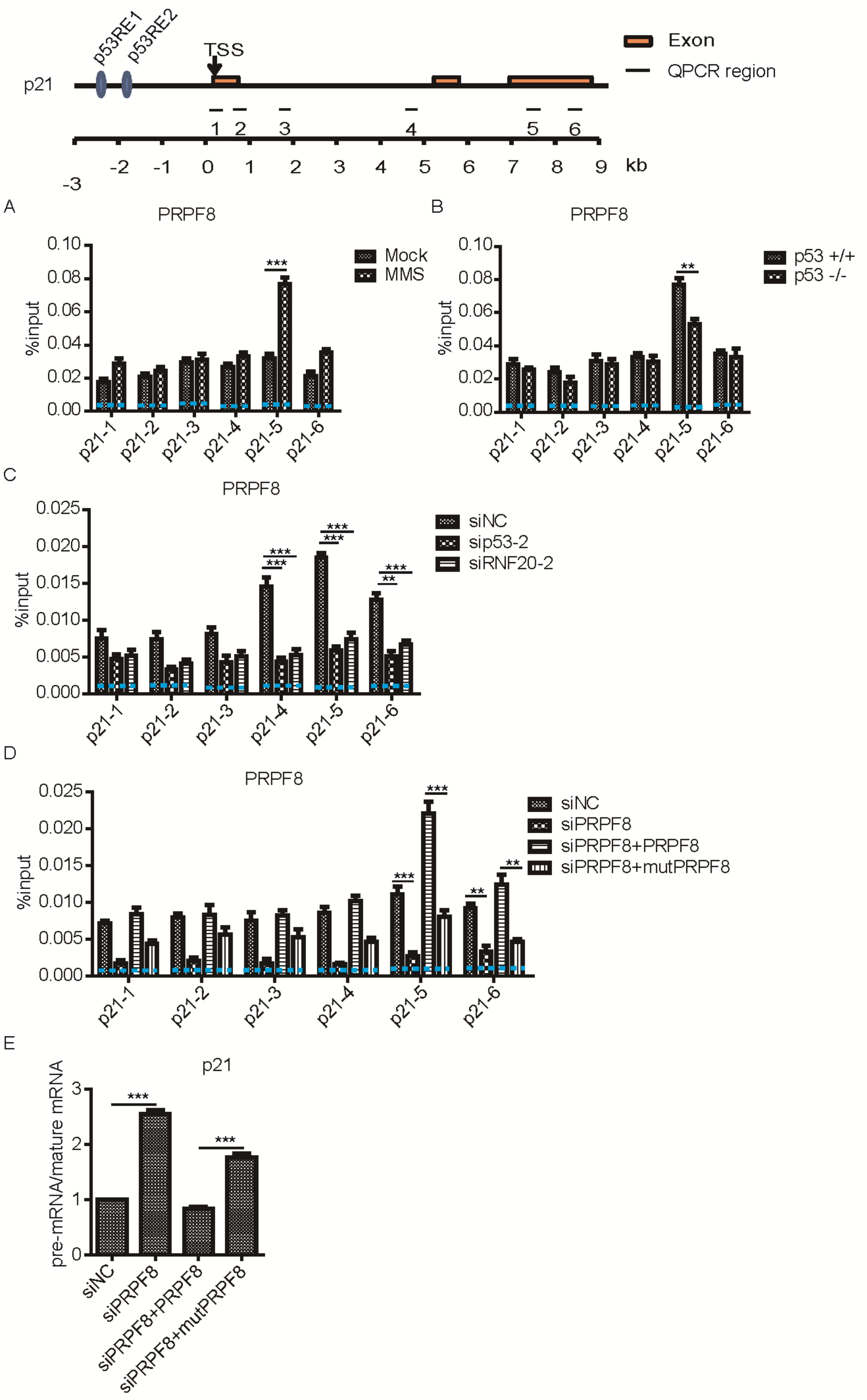

Supplementary Figure S11. PRPF8 impairs the pre-mRNA processing of *p21* in HCT116 cells.

(A) PRPF8 is recruited to the gene body of *p21* upon MMS induced DNA damage. HCT116

cells were treated with or without 2 mM MMS. ChIP analyses at the *p21* locus were performed using anti-PRPF8 antibodies. An irrelevant IgG was used for negative control presented as the dotted lines. Primers used for q-PCR are included in Supplemental Table S3. (B) p53-/- HCT116 cells were used in ChIP assay as a control for p53+/+ HCT116. (C) Downregulation of p53 or the RNF20/40 complex by another siRNA impairs the enrichment of PRPF8 at the gene body region of *p21* in HCT116 cells. (D) The I2105A/I2106A mutant of PRPF8 (mutPRPF8) impairs the enrichment of PRPF8 at the gene body region of *p21* in response to MMS. (E) MutPRPF8 impairs the pre-mRNA processing of *p21*. HCT116 cells were treated with indicated siRNA. Full length PRPF8 or mutPRPF8 were re-introduced into the cells. The cells were treated with 2 mM MMS. The relative changes of pre-mRNA vs. mature mRNA of *p21* were examined by q-PCR. The primer sequences have been included in Supplemental Table S5. Data are represented as mean ± SD as indicated from three independent experiments. *: Statistically Significant ($p < 0.05$); **: Statistically Significant ($p < 0.01$); ***: Statistically Significant ($p < 0.001$).

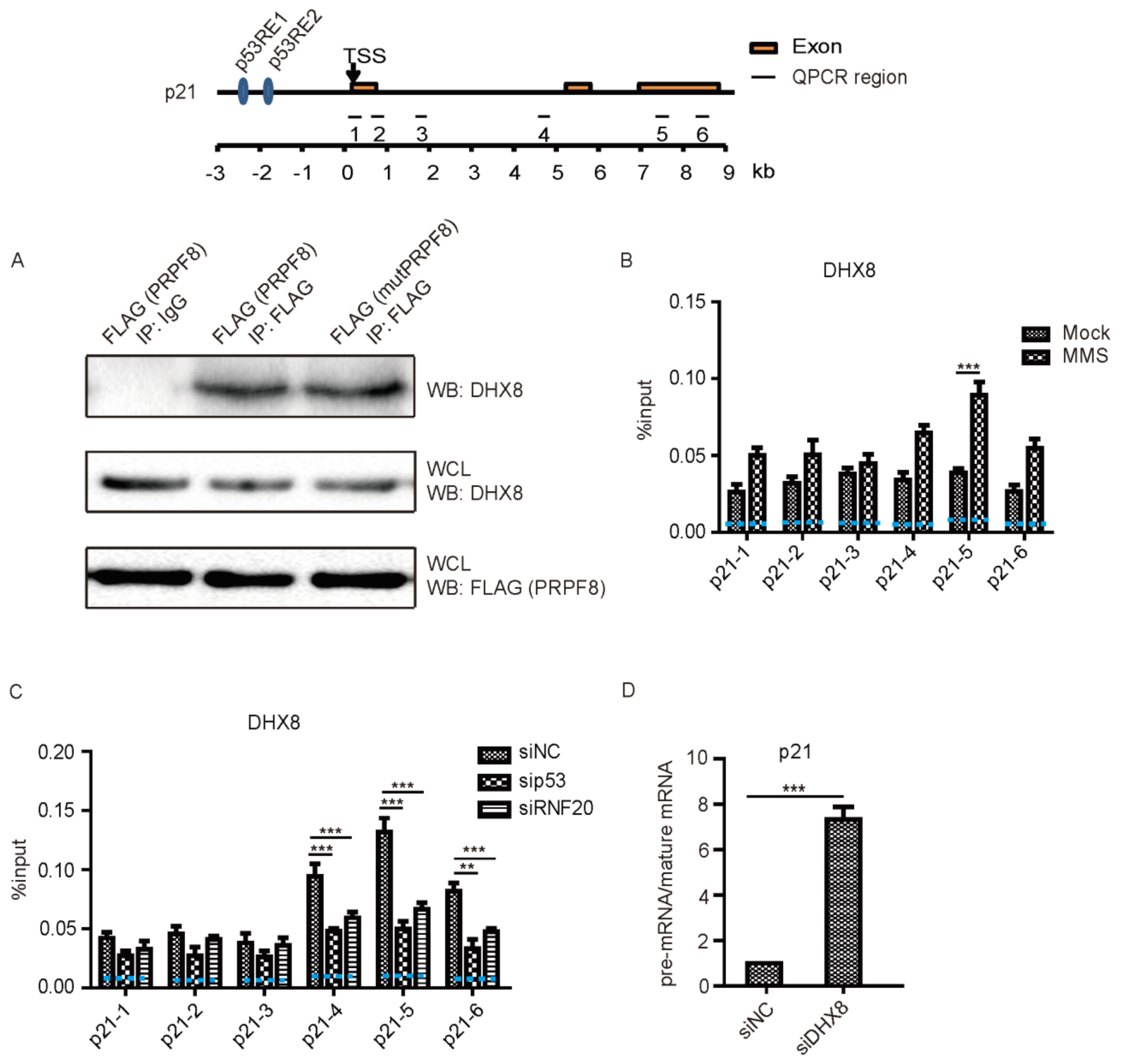

Supplementary Figure S12. DHX8 impairs the pre-mRNA processing of *p21* in HCT116 cells.

(A) Both of PRPF8 and mutPRPF8 associate with endogenous DHX8. HCT116 cells were transfected with FLAG-PRPF8 or FLAG-mut PRPF8. Cells were lyzed with NETN300 buffer. Cell extracts were examined by Co-IP and Western blot with indicated antibodies using unspecific IgG as a negative control. (B) DHX8 is recruited to the gene body of *p21* upon DNA damage. HCT116 cells were treated with or without 2mM MMS. ChIP analyses at the *p21* locus were performed using anti-DHX8 antibodies. An irrelevant IgG was used for

negative control presented as the dotted lines. Primers used for q-PCR are included in Supplemental Table S3. (C) Downregulation of p53 or the RNF20/40 complex by siRNA impairs the enrichment of DHX8 at the gene body region of *p21* in HCT116 cells. (D) DHX8 impairs the pre-mRNA processing of *p21*. HCT116 cells were treated with indicated siRNA. The cells were treated with 2mM MMS. The relative changes of pre-mRNA vs. mature mRNA of *p21* were examined by q-PCR. The primer sequences have been included in Supplemental Table S5. Data are represented as mean ±SD as indicated from three independent experiments. *: Statistically Significant ($p < 0.05$); **: Statistically Significant ($p < 0.01$); ***: Statistically Significant ($p < 0.001$). (D) Both of PRPF8 and mutPRPF8 associate with endogenous DHX8. cells were lyzed with NETN300 buffer, and lysates were examined by IP and Western blot with indicated antibodies. GAPDH was used as the protein loading controls.

II. Supplementary tables

Supplementary Table S1. Affinaty purification and mass spectrometry analysis of p53 in HCT116 cell.

GYGI LAB / TAPLIN BIOLOGICAL MASS SPEC FACILITY SearchWiz | Status | Browse

/home/www/production/html/saved_sets/12870.2.sumlist

406 peptides, **94** proteins Charge states: 1+ ☑ 2+ ☑ 3+ ☑ 4+ ☑ Sort: Reference desc ◉ html ○ text ○ dta Peptides Reload

12870.2 Lookup ○ peptide ◉ reference ☐ Group 2/3+ ☐ Use best ion

scanf, scanl, Rank, Rank_Sp, charge, Ions, premass, _M_H_, mass_diff

R: ☐ H: ☐ Sequence:
R: ☐ H: ☐ Peptide type: tryptic
R: ☐ H: ☐ AA type: acidic
R: ☐ H: ☐ Mod AA type: acidic

XCorr 1+ 0 XCorr 2+ 0 XCorr 3+ 0 XCorr 4+ 0 dCn: dCn2:

Rank (1 to n): Low PPM1: High PPM1: Low CalPPM: High CalPPM: Vista Score:

☐ Hide duplicate peptides

Protein matches					☐	scanf	Rank	charge	Ions	dCn	dCn2	XCorr	Reference	Redu	Peptide
67	67	3.754	0.00	UPSP:PARC_HUMAN	☐	2686	1	2	24/36	0.5097	0.5097	5.1340	UPSP:PARC_HUMAN	6	K.ELFIEDPSPAISILVLSPR.C
58	58	3.512	0.00	UPSP:CUL7_HUMAN	☐	1892	1	2	24/26	0.3775	0.3775	5.2095	UPSP:PARC_HUMAN	6	K.FNQTEEVSVETLLK.D
43	43	3.632	0.00	UPSP:COA1_HUMAN	☐	2774	1	3	36/132	0.6037	0.6037	5.4201	UPSP:PARC_HUMAN	4	K.GAGATVLGTAFPSWDWNPM*I
20	20	3.226	0.00	UPSP:P53_HUMAN	☐	0826	1	2	21/28	0.3057	0.3057	3.0963	UPSP:PARC_HUMAN	5	K.GLQHEPAGVSGSFPR.D
13	13	3.805	0.00	UPSP:BRE1A_HUMAN	☐	1913	1	2	11/12	0.0615	0.0615	2.0079	UPSP:PARC_HUMAN	5	K.LLFSLVK.R
11	11	3.989	0.00	UPSP:MCCC2_HUMAN	☐	0474	1	2	11/12	0.1001	0.1001	2.5396	UPSP:PARC_HUMAN	5	K.LYEHLQR.A
11	11	3.595	0.00	UPSP:NUCL_HUMAN	☐	1758	1	2	23/32	0.3173	0.3173	5.0465	UPSP:PARC_HUMAN	5	K.M*LAVASSSEIPTFVTGR.D
8	8	4.158	0.00	UPSP:MCCA_HUMAN	☐	0551	1	2	22/24	0.3530	0.3530	4.2118	UPSP:PARC_HUMAN	5	K.M*LQALAAHDAGSR.A
8	8	3.784	0.00	UPSP:BRE1B_HUMAN	☐	1589	1	2	22/24	0.4172	0.4172	4.9854	UPSP:PARC_HUMAN	4	K.M*LVELLTNQVGEK.M
7	7	3.410	0.00	UPSP:FBXW8_HUMAN	☐	0768	1	2	13/14	0.3182	0.3182	2.6531	UPSP:PARC_HUMAN	4	K.M*VVVQALR.L
6	6	3.861	0.00	UPSP:PYC_HUMAN	☐	1947	1	2	23/32	0.5213	0.5213	4.7461	UPSP:PARC_HUMAN	5	K.MLAVASSSEIPTFVTGR.D
6	6	3.557	0.00	UPSP:HNRPU_HUMAN	☐	1897	1	2	21/24	0.4335	0.4335	4.7777	UPSP:PARC_HUMAN	4	K.MLVELLTNQVGEK.M
6	6	2.892	0.00	UPSP:RS2_HUMAN	☐	1094	1	2	14/14	0.0998	0.0998	1.9294	UPSP:PARC_HUMAN	4	K.MVVVQALR.L
5	5	4.181	0.00	UPTR:Q59FY4_HUMAN	☐	0652	1	2	14/16	0.1137	0.1137	2.3118	UPSP:PARC_HUMAN	3	K.NLDETLGEK.A
5	5	4.027	0.00	UPSP:HSP7C_HUMAN	☐	2270	1	3	27/56	0.3364	0.3364	3.9926	UPSP:PARC_HUMAN	6	K.QILHVSTVQM*WLLLK.F
5	5	3.912	0.00	UPSP:RLA0_HUMAN	☐	1956	1	2	13/14	0.1423	0.1423	2.5488	UPSP:PARC_HUMAN	5	K.TLLLSVLR.V
5	5	3.854	0.00	UPSP:DECR_HUMAN	☐	2157	1	3	41/120	0.5460	0.5460	5.7755	UPSP:PARC_HUMAN	6	K.TRTETPM*AQSDSQLFNQLLVI
5	5	3.635	0.00	UPSP:RL7A_HUMAN	☐	1579	1	2	22/26	0.3391	0.3391	4.5833	UPSP:PARC_HUMAN	5	K.TSVLVQQAGLAALK.M
4	4	4.267	0.00	UPSP:NPM_HUMAN	☐	0896	1	2	18/28	0.3943	0.3943	3.6828	UPSP:PARC_HUMAN	6	K.YGLLSNEPSSSSTSR.N
4	4	3.858	0.00	UPTR:Q7Z561_HUMAN	☐	1489	1	2	13/14	0.1760	0.1760	3.1445	UPSP:PARC_HUMAN	5	R.AAFM*LALR.S
4	4	3.570	0.00	UPTR:Q6NXR8_HUMAN	☐	2036	1	2	27/38	0.6230	0.6230	5.5953	UPSP:PARC_HUMAN	5	R.AALETPIIQGQDGSPELLIR.S
4	4	3.565	0.00	UPSP:RL3_HUMAN	☐	0709	1	2	10/12	0.1102	0.1102	2.3493	UPSP:PARC_HUMAN	6	R.AELQFGK.Q
3	3	3.674	0.00	UPTR:Q53HV1_HUMAN	☐	0908	1	2	14/16	0.2005	0.2005	2.7715	UPSP:PARC_HUMAN	5	R.AGSELFGPR.A
3	3	3.646	0.00	UPSP:PCCB_HUMAN	☐	1362	1	3	35/84	0.4415	0.4415	5.2161	UPSP:PARC_HUMAN	5	R.AHVLLSLSQQDGIEQHM*DFDS
3	3	3.580	0.00	UPSP:HSP71_HUMAN	☐	1691	1	3	31/84	0.2020	0.2020	3.3432	UPSP:PARC_HUMAN	5	R.AHVLLSLSQQDGIEQHMDFDSI
3	3	3.272	0.00	UPSP:ECHA_HUMAN	☐	0866	1	2	15/20	0.2777	0.2777	2.9497	UPSP:PARC_HUMAN	6	R.AIVSSPEVISK.Y
3	3	3.260	0.00	UPSP:RL4_HUMAN	☐	0777	1	2	11/12	0.2084	0.2084	2.3872	UPSP:PARC_HUMAN	5	R.APDFVPR.Y
3	3	2.985	0.00	UPSP:RS24_HUMAN	☐	1875	1	3	37/84	0.3394	0.3394	3.7283	UPSP:PARC_HUMAN	5	R.DPGGLDEVAM*GEM*EADVQA
3	3	2.972	0.00	UPSP:H12_HUMAN	☐	2448	1	2	28/42	0.1611	0.5789	6.0230	UPSP:PARC_HUMAN	5	R.DPGGLDEVAM*GEMEADVQAI
2	2	4.698	0.00	UPSP:RL6_HUMAN	☐	1040	1	2	21/30	0.3137	0.3137	4.2542	UPSP:PARC_HUMAN	5	R.DRSPAPSPVLPSSSLR.N
2	2	4.546	0.00	UPSP:TBA6_HUMAN	☐	1301	1	3	28/48	0.3355	0.3355	3.9003	UPSP:PARC_HUMAN	5	R.DSIHSLFDAQM*TR.E
2	2	4.427	0.00	UPSP:TBA2_HUMAN	☐	1118	1	2	8/12	0.0295	0.0295	1.7661	UPSP:PARC_HUMAN	6	R.EFAVNLR.N
2	2	4.410	0.00	UPSP:GRP78_HUMAN	☐	0758	1	2	14/24	0.3043	0.3043	3.0644	UPSP:PARC_HUMAN	5	R.EGSPGGAVRPLLK.R
2	2	4.378	0.00	UPTR:Q96FW6_HUMAN	☐	2133	1	2	22/32	0.5575	0.5575	4.2857	UPSP:PARC_HUMAN	6	R.FEGSTLNDLLNSQIYTK.Y
2	2	4.218	0.00	UPSP:G3P_HUMAN	☐	1299	1	2	11/12	0.1934	0.1934	2.4670	UPSP:PARC_HUMAN	5	R.FLAAAWR.A
2	2	3.875	0.00	UPSP:RL7_HUMAN	☐	1065	1	3	35/76	0.4500	0.4500	4.2533	UPSP:PARC_HUMAN	6	R.FSSFYSQSQNHPVLDM*GPHR.I
2	2	3.661	0.00	UPTR:Q9BS10_HUMAN	☐	2433	1	3	27/60	0.4729	0.4729	4.5919	UPSP:PARC_HUMAN	5	R.GVEVLGPKPTFWPLFR.E
2	2	3.620	0.00	UPTR:Q6IPX9_HUMAN	☐	1007	1	2	15/22	0.4728	0.4728	2.9748	UPSP:PARC_HUMAN	5	R.HAGDLM*VPLGPR.L
2	2	3.554	0.00	UPSP:RL21_HUMAN	☐	1681	1	2	21/24	0.4381	0.4381	4.1353	UPSP:PARC_HUMAN	6	R.LLAILQHSAQDFR.V
2	2	3.378	0.00	UPSP:SYR_HUMAN	☐	1663	1	2	10/14	0.0340	0.0340	1.9905	UPSP:PARC_HUMAN	4	R.LLYLLM*TK.H
2	2	3.248	0.00	UPTR:Q59FI9_HUMAN	☐	1851	1	2	27/32	0.4986	0.4986	5.7242	UPSP:PARC_HUMAN	6	R.LPQLM*LQSLSTSEELQR.Q
2	2	3.035	0.00	UPTR:Q9NQS8_HUMAN	☐	2111	1	2	24/32	0.4936	0.4936	5.6182	UPSP:PARC_HUMAN	6	R.LPQLMLQSLSTSEELQR.Q
2	2	2.936	0.00	GP:M77233_1	☐	1487	1	2	15/18	0.1706	0.1706	2.4245	UPSP:PARC_HUMAN	5	R.LQAYPEELIR.Q
2	2	2.663	0.00	UPSP:RS9_HUMAN	☐	2461	1	2	21/24	0.3515	0.3515	5.0070	UPSP:PARC_HUMAN	5	R.LQQETQPFLLLLR.T
1	1	6.738	0.00	UPTR:Q6LC01_HUMAN	☐	2028	1	2	11/14	0.1161	0.1161	2.3831	UPSP:PARC_HUMAN	6	R.LQWTWLGR.A
1	1	5.678	0.00	UPSP:TCPE_HUMAN	☐	1593	1	3	19/32	0.1115	0.1115	3.0142	UPSP:PARC_HUMAN	6	R.QFHLFQLQR.L
1	1	5.396	0.00	UPSP:AP2B1_HUMAN	☐	2137	1	2	15/34	0.1993	0.1993	2.3199	UPSP:PARC_HUMAN	4	R.QSNNGIPPVQVFWQSTGR.T
1	1	5.307	0.00	UPSP:MAGB2_HUMAN	☐	0926	1	2	10/12	0.1182	0.1182	2.1793	UPSP:PARC_HUMAN	5	R.RQGWVFR.Q
1	1	5.195	0.00	UPSP:RS6_HUMAN	☐	2516	1	2	19/34	0.3608	0.3608	3.4220	UPSP:PARC_HUMAN	5	R.SGEALWLIPPQAYLNVEK.D
1	1	4.952	0.00	UPSP:RL14_HUMAN	☐	2384	1	2	18/42	0.3999	0.3999	3.8902	UPSP:PARC_HUMAN	5	R.SGEALWLIPPQAYLNVEKDEGI
1	1	4.905	0.00	UPSP:RL9_HUMAN	☐	2363	1	3	30/92	0.5139	0.5139	4.3534	UPSP:PARC_HUMAN	5	R.SGFSGALLQQSFLTAAHM*SEQ
1	1	4.742	0.00	UPTR:Q6NZ54_HUMAN	☐	1256	1	2	19/30	0.4830	0.4830	4.5462	UPSP:PARC_HUMAN	5	R.SGYGEYVQQTLQPGM*R.V

1	1	4.645	0.00	UPTR:O75147_HUMAN	☐	1417	1	2	18/30	0.4068	0.4068	4.1901	UPSP:PARC_HUMAN	5	R.SGYGEYVQQTLQPGMR.V
1	1	4.612	0.00	UPSP:SDPR_HUMAN	☐	2694	1	2	27/42	0.4596	0.4596	5.9261	UPSP:PARC_HUMAN	5	R.SIFQPYISGPSLLLPTIVTTPR.R
1	1	4.588	0.00	UPSP:LA_HUMAN	☐	2399	1	2	22/30	0.5176	0.5176	4.8656	UPSP:PARC_HUMAN	5	R.SLVGGPSAELLLDLER.V
1	1	4.567	0.00	UPSP:RL10_HUMAN	☐	1102	1	2	15/26	0.3395	0.3395	2.8862	UPSP:PARC_HUMAN	5	R.SPAPSPVLPSSSLR.N
1	1	4.538	0.00	UPTR:Q5JR95_HUMAN	☐	2743	1	2	22/28	0.4458	0.4458	4.2121	UPSP:PARC_HUMAN	6	R.THQPINIPFFDVFLR.H
1	1	4.478	0.00	UPSP:RS17_HUMAN	☐	1984	1	2	15/16	0.2533	0.2533	2.5277	UPSP:PARC_HUMAN	5	R.TILM*M*LLNR.Y
1	1	4.405	0.00	UPTR:Q6ICQ4_HUMAN	☐	2355	1	2	14/16	0.1423	0.2890	2.5961	UPSP:PARC_HUMAN	5	R.TILM*MLLNR.Y
1	1	4.402	0.00	GP:X60020_1	☐	0392	1	2	13/16	0.2646	0.2646	2.4141	UPSP:PARC_HUMAN	5	R.TLDAPGPNK.T
1	1	4.319	0.00	UPSP:MDM2_HUMAN	☐	0643	1	2	17/22	0.3606	0.3606	3.4825	UPSP:PARC_HUMAN	6	R.TM*SPQEVEGLM*K.Q
1	1	4.301	0.00	UPSP:COA2_HUMAN	☐	1116	1	2	15/22	0.2830	0.2830	2.4627	UPSP:PARC_HUMAN	6	R.TM*SPQEVEGLMK.Q
1	1	4.270	0.00	UPTR:Q59FS3_HUMAN	☐	1461	1	2	18/26	0.5004	0.5004	5.1485	UPSP:PARC_HUMAN	6	R.VGLQSPSVEAWEAK.G
1	1	4.235	0.00	UPTR:Q53G49_HUMAN	☐	1514	1	2	15/16	0.2018	0.2018	3.1822	UPSP:PARC_HUMAN	4	R.VILLENLNR.F
1	1	4.166	0.00	UPSP:IF2G_HUMAN	☐	0562	1	2	18/20	0.4409	0.4409	3.8727	UPSP:PARC_HUMAN	6	R.VSAIHEVPPPR.S
1	1	4.087	0.00	UPSP:DDX1_HUMAN	☐	2022	1	3	37/96	0.3624	0.3624	4.7703	UPSP:PARC_HUMAN	5	R.YIDQQIQGGLIGGAPGVEM*LG
1	1	4.082	0.00	UPSP:DBPA_HUMAN	☐	2348	1	3	34/96	0.3643	0.3643	4.2653	UPSP:PARC_HUMAN	5	R.YIDQQIQGGLIGGAPGVEMLG
1	1	4.054	0.00	UPSP:H2B1B_HUMAN	☐	0462	1	2	16/18	0.3093	0.3093	2.9897	UPSP:CUL7_HUMAN	2	K.AAHVSEQFAR.H
1	1	4.007	0.00	UPTR:Q6PB27_HUMAN	☐	0544	1	2	16/24	0.3670	0.3670	2.6332	UPSP:CUL7_HUMAN	2	K.EPPSQSPNTPLQR.L
1	1	3.998	0.00	UPSP:TCPD_HUMAN	☐	0543	1	2	12/12	0.2714	0.2714	2.4605	UPSP:CUL7_HUMAN	2	K.HLDFDSR.C
1	1	3.985	0.00	UPSP:SYD_HUMAN	☐	2122	1	3	33/68	0.4324	0.4324	4.6642	UPSP:CUL7_HUMAN	2	K.ILLDLEQALSSEGTQENK.V
1	1	3.880	0.00	UPTR:Q6IRZ0_HUMAN	☐	0705	1	1	13/18	0.2454	0.2454	2.2170	UPSP:CUL7_HUMAN	2	K.IQVGLGASGK.E
1	1	3.879	0.00	UPSP:HSP72_HUMAN	☐	0584	1	2	16/20	0.3596	0.3596	3.0684	UPSP:CUL7_HUMAN	2	K.KIQVGLGASGK.E
1	1	3.840	0.00	UPTR:Q5VTE0_HUMAN	☐	2500	1	3	35/128	0.3877	0.3877	4.2002	UPSP:CUL7_HUMAN	2	K.KYGPEALAGNQAYPSLLEAQE
1	1	3.765	0.00	UPSP:KC1AL_HUMAN	☐	1229	1	2	13/14	0.0853	0.0853	3.3701	UPSP:CUL7_HUMAN	2	K.LELAQELR.D
1	1	3.671	0.00	UPSP:HSP76_HUMAN	☐	1318	1	2	28/46	0.5589	0.5589	6.0018	UPSP:CUL7_HUMAN	2	K.M*LGEDGQVIGPSQESAGEVG
1	1	3.620	0.00	UPTR:Q1JQ76_HUMAN	☐	1455	1	2	27/46	0.4827	0.4827	5.6671	UPSP:CUL7_HUMAN	2	K.MLGEDGQVIGPSQESAGEVGA
1	1	3.597	0.00	UPSP:HNRPQ_HUMAN	☐	1686	1	2	18/20	0.3318	0.3318	3.2310	UPSP:CUL7_HUMAN	2	K.QVNNFLTSSWR.D
1	1	3.588	0.00	UPTR:Q5JP53_HUMAN	☐	1853	1	3	46/132	0.4937	0.4937	7.6666	UPSP:CUL7_HUMAN	2	K.RYLHVTSLLDQLNDSAAEPGA
1	1	3.577	0.00	GP:AE006463_8	☐	0668	1	2	17/20	0.4314	0.4314	3.9138	UPSP:CUL7_HUMAN	2	K.SVLEEM*ETDVK.S
1	1	3.557	0.00	UPSP:HS90A_HUMAN	☐	1529	1	2	14/20	0.3131	0.3131	2.6216	UPSP:CUL7_HUMAN	2	K.SVLEEMETDVK.S
1	1	3.500	0.00	UPSP:MDM4_HUMAN	☐	1471	1	2	17/18	0.3872	0.3872	3.2581	UPSP:CUL7_HUMAN	2	K.VKPLLLQLQR.Q
1	1	3.499	0.00	UPSP:HS70L_HUMAN	☐	2618	1	3	43/124	0.5660	0.5660	5.5243	UPSP:CUL7_HUMAN	2	K.YGPEALAGNQAYPSLLEAQED
1	1	3.497	0.00	UPSP:PCCA_HUMAN	☐	0718	1	2	11/12	0.0766	0.0766	1.8806	UPSP:CUL7_HUMAN	2	R.DDDFVPR.Y
1	1	3.478	0.00	UPSP:TCPB_HUMAN	☐	0112	1	2	11/14	0.1829	0.1829	2.0937	UPSP:CUL7_HUMAN	2	R.DTLQPGM*R.V
1	1	3.362	0.00	UPSP:HNRPD_HUMAN	☐	1687	1	2	17/22	0.3633	0.3633	3.9173	UPSP:CUL7_HUMAN	2	R.DYAVVLNQLGAR.D
1	1	3.361	0.00	UPTR:Q5VVC8_HUMAN	☐	2680	1	2	17/22	0.1137	0.1137	4.2781	UPSP:CUL7_HUMAN	2	R.EVLQELIFFLHR.L
1	1	3.357	0.00	UPTR:Q53EN9_HUMAN	☐	1296	1	2	21/24	0.3061	0.3061	3.5881	UPSP:CUL7_HUMAN	8	R.FLPDDEAAQALGK.T
1	1	3.351	0.00	UPSP:RL18_HUMAN	☐	2265	1	2	12/14	0.0196	0.0196	2.4518	UPSP:CUL7_HUMAN	7	R.FWPIIQIR.I
1	1	3.102	0.00	UPSP:SYK_HUMAN	☐	2517	1	2	17/30	0.4389	0.4389	3.4029	UPSP:CUL7_HUMAN	2	R.GLEILGPKPTFWPVFR.E
1	1	3.101	0.00	UPTR:Q5JWB0_HUMAN	☐	0733	1	1	7/14	0.1630	0.1630	1.6557	UPSP:CUL7_HUMAN	2	R.GLVSSLGK.G
1	1	3.077	0.00	UPSP:RL13_HUMAN	☐	0984	1	2	10/12	0.1885	0.1885	2.2211	UPSP:CUL7_HUMAN	2	R.GPAFFSR.V
1	1	3.069	0.00	GP:AY891881_1	☐	0569	1	2	14/16	0.3823	0.3823	3.4820	UPSP:CUL7_HUMAN	2	R.GPLDLHEQK.D
1	1	2.814	0.00	UPSP:DHX9_HUMAN	☐	1294	1	3	24/64	0.4060	0.4060	3.8084	UPSP:CUL7_HUMAN	2	R.GPLDLHEQKDIPGGVLK.I
						2769	1	3	28/88	0.4079	0.4079	4.3136	UPSP:CUL7_HUMAN	2	R.GQLELEFSM*AM*GTLISELVQ.
						0319	1	2	17/18	0.1840	0.1840	3.6296	UPSP:CUL7_HUMAN	2	R.HIDQQIQGSR.I
						1552	1	2	23/38	0.3746	0.3746	2.7451	UPSP:CUL7_HUMAN	2	R.LIPPQTYLQAEGEDGQNLEK.R
						0452	1	2	12/12	0.1748	0.1748	2.3680	UPSP:CUL7_HUMAN	2	R.LNDSALR.D
						0898	1	2	13/24	0.3210	0.3210	2.6753	UPSP:CUL7_HUMAN	2	R.LPQQM*LQSLSTSK.E
						1454	1	2	19/24	0.3823	0.3823	3.9411	UPSP:CUL7_HUMAN	2	R.LPQQMLQSLSTSK.E
						0590	1	2	15/18	0.3687	0.3687	3.0308	UPSP:CUL7_HUMAN	2	R.LVEGYGPAGK.I
						0498	1	2	20/24	0.4497	0.4497	3.7290	UPSP:CUL7_HUMAN	2	R.M*IQALSSHDAGTR.T
						1446	1	3	31/64	0.3622	0.3622	3.7057	UPSP:CUL7_HUMAN	8	R.M*LDDYEEISAGDEGEFR.Q
						1728	1	2	15/32	0.2050	0.2050	2.7548	UPSP:CUL7_HUMAN	8	R.MLDDYEEISAGDEGEFR.Q
						0111	1	2	11/14	0.0801	0.0801	1.6180	UPSP:CUL7_HUMAN	2	R.QEQNFADR.F
						2174	1	3	27/60	0.2506	0.2506	4.4303	UPSP:CUL7_HUMAN	2	R.QFHVYQLQQLDQELLK.L
						1667	1	2	23/32	0.4157	0.4157	5.1650	UPSP:CUL7_HUMAN	7	R.QLTLLVASEDSSYM*PAR.V
						1868	1	2	24/32	0.4878	0.4878	4.6842	UPSP:CUL7_HUMAN	7	R.QLTLLVASEDSSYMPAR.V
						2090	1	3	29/72	0.3164	0.3164	4.3630	UPSP:CUL7_HUMAN	2	R.QPQPFLALM*QSLDTPETNR.T
						2633	1	3	25/72	0.3571	0.3571	3.9194	UPSP:CUL7_HUMAN	2	R.QPQPFLALMQSLDTPETNR.T
						2064	1	2	21/34	0.4861	0.4861	3.2180	UPSP:CUL7_HUMAN	2	R.QSNNGVPPVQVFWESTGR.T
						1479	1	2	20/26	0.4326	0.4326	3.3158	UPSP:CUL7_HUMAN	2	R.SEFASGNTYALYVR.D
						0751	1	2	15/18	0.4407	0.4407	2.6660	UPSP:CUL7_HUMAN	2	R.SHDWSSLATR.G
						2139	1	3	34/104	0.4918	0.4918	4.9262	UPSP:CUL7_HUMAN	2	R.SPGSIFQPQLADVSPGLPAAQA
						2622	1	3	32/56	0.3498	0.3498	4.1034	UPSP:CUL7_HUMAN	1	R.THRPINIPFFDVFLR.Y
						1159	1	2	13/14	0.0698	0.0698	2.0158	UPSP:CUL7_HUMAN	3	R.TLHLTVLR.I
						1854	1	2	22/28	0.3449	0.3449	3.9829	UPSP:CUL7_HUMAN	2	R.TQILLSLSQQEAIEK.H
						0475	1	2	15/22	0.2975	0.2975	2.9008	UPSP:CUL7_HUMAN	2	R.VGHDGHPEYQIR.W

1714	1	2	15/16	0.2144	0.2144	2.9832	UPSP:CUL7_HUMAN	2	R.VILLENLTR.F
1890	1	2	17/32	0.3795	0.3795	3.3189	UPSP:CUL7_HUMAN	2	R.VLDLLM*HM*LSSPDYQIR.W
2424	1	2	13/32	0.2230	0.2786	2.5445	UPSP:CUL7_HUMAN	2	R.VLDLLM*HMLSSPDYQIR.W
1631	1	2	11/12	0.0764	0.0764	2.2139	UPSP:CUL7_HUMAN	2	R.VLFSLVK.R
1694	1	3	31/60	0.5287	0.5287	4.4522	UPSP:CUL7_HUMAN	2	R.VPLGPGLHAYPDELIR.Q
2034	1	3	38/128	0.4010	0.4010	5.5083	UPSP:CUL7_HUMAN	2	R.YLHVTSLLDQLNDSAAEPGAQ[illegible]
0560	1	2	11/12	0.2027	0.2027	2.2073	UPSP:CUL7_HUMAN	2	R.YSNFYNK.S
1268	1	2	13/14	0.1682	0.1682	1.9468	UPSP:COA1_HUMAN	2	K.AVVM*DLLR.Q
2171	1	3	28/60	0.3077	0.3077	4.6556	UPSP:COA1_HUMAN	4	K.AYVWDNNKDLAEWLEK.Q
1651	1	2	13/18	0.1298	0.1298	2.3804	UPSP:COA1_HUMAN	9	K.FGAYIVDGLR.E
2317	1	2	21/44	0.5864	0.5864	4.1657	UPSP:COA1_HUMAN	4	K.IASSIVAQTAGIPTLPWSGSGLR
1430	1	3	33/64	0.3178	0.3178	4.5948	UPSP:COA1_HUMAN	4	K.ITDIIGKEEGIGPENLR.G
0899	1	2	17/20	0.3498	0.3498	3.5291	UPSP:COA1_HUMAN	2	K.LLETESFQM*NR.I
1627	1	2	10/12	0.1226	0.1226	2.2766	UPSP:COA1_HUMAN	4	K.LPELLLK.N
0601	1	2	13/16	0.0081	0.0081	2.0510	UPSP:COA1_HUMAN	10	K.M*HLYLGAAK.V
2160	1	3	26/76	0.3040	0.3040	3.0787	UPSP:COA1_HUMAN	3	K.RFQAQSLGTTYIYDIPEM*FR.Q
1611	1	3	35/72	0.4915	0.4915	5.6128	UPSP:COA1_HUMAN	8	K.RIPVQAVWAGWGHASENPK.L
2315	1	3	31/68	0.3985	0.3985	5.0553	UPSP:COA1_HUMAN	2	K.SDM*NTVLNYIFSHAQVTK.K
2379	1	3	33/84	0.2247	0.2247	3.9211	UPSP:COA1_HUMAN	2	K.TLRDPSLPLLELQDIM*TSVSG[illegible]
2792	1	3	34/84	0.4325	0.4325	5.1788	UPSP:COA1_HUMAN	2	K.TLRDPSLPLLELQDIMTSVSGR.
0879	1	2	18/20	0.4212	0.4212	4.0077	UPSP:COA1_HUMAN	3	K.VEVGTEVTDYR.F
1235	1	2	18/22	0.0406	0.0406	2.2645	UPSP:COA1_HUMAN	9	K.VLIANNGIAAVK.C
1740	1	2	16/18	0.2686	0.2686	3.4959	UPSP:COA1_HUMAN	11	R.AIGIGAYLVR.L
1816	1	2	18/24	0.4147	0.4147	4.2008	UPSP:COA1_HUMAN	11	R.DFTVASPAEFVTR.F
2452	1	2	14/22	0.4198	0.4198	3.1998	UPSP:COA1_HUMAN	4	R.EGLPLM*VFANWR.G
2249	1	3	25/60	0.2788	0.2788	3.4694	UPSP:COA1_HUMAN	2	R.EVFFM*NTQSIVQLVQR.Y
2556	1	2	20/30	0.2182	0.2182	4.6371	UPSP:COA1_HUMAN	2	R.EVFFMNTQSIVQLVQR.Y
2081	1	2	27/38	0.4769	0.4769	6.1663	UPSP:COA1_HUMAN	5	R.FIIGSVSEDNSEDEISNLVK.L
2321	1	2	21/36	0.5900	0.5900	5.4766	UPSP:COA1_HUMAN	3	R.FQAQSLGTTYIYDIPEM*FR.Q
1539	1	2	16/20	0.2190	0.2190	3.0436	UPSP:COA1_HUMAN	8	R.FVVM*VTPEDLK.A
2470	1	3	32/88	0.4961	0.4961	5.2292	UPSP:COA1_HUMAN	2	R.GQVLPAHTLLNTVDVELIYEG[illegible]
1202	1	2	19/24	0.2282	0.2282	3.8678	UPSP:COA1_HUMAN	4	R.GSVLEPEGTVEIK.F
1048	1	2	13/14	0.2280	0.2280	2.8145	UPSP:COA1_HUMAN	4	R.IGLAEEIR.H
2343	1	2	21/26	0.3880	0.3880	4.2474	UPSP:COA1_HUMAN	4	R.IGSFGPQEDLLFLR.A
1275	1	2	13/14	0.0445	0.0445	2.3492	UPSP:COA1_HUMAN	4	R.IIEFVPTK.T
1502	1	2	17/20	0.2153	0.2153	3.1323	UPSP:COA1_HUMAN	4	R.ILNVPQELYEK.G
1777	1	3	26/68	0.0081	0.0081	3.0134	UPSP:COA1_HUMAN	8	R.IPVQAVWAGWGHASENPK.L
1433	1	3	31/88	0.4357	0.4357	4.4520	UPSP:COA1_HUMAN	7	R.ITSENPDEGFKPSSGTVQELNF[illegible]
1649	1	2	19/26	0.4212	0.4212	2.9607	UPSP:COA1_HUMAN	4	R.LGGIPVGVVAVETR.T
1463	1	2	19/24	0.4194	0.4194	4.2185	UPSP:COA1_HUMAN	2	R.LPGGNEIGM*VAWK.M
1734	1	3	26/96	0.1532	0.1532	3.5448	UPSP:COA1_HUMAN	2	R.LSDGGLLLSYDGSSYTTYM*K[illegible]
1110	1	2	13/16	0.1878	0.1878	2.6893	UPSP:COA1_HUMAN	2	R.M*AALEVYVR.R
0520	1	2	12/14	0.1688	0.1688	2.0494	UPSP:COA1_HUMAN	2	R.M*GGM*VSFR.T
1932	1	2	16/22	0.3791	0.3791	2.9207	UPSP:COA1_HUMAN	2	R.NSVSNFLHSLER.G
1490	1	2	19/28	0.2675	0.2675	3.9247	UPSP:COA1_HUMAN	2	R.QVQAEVPGSPIFVM*R.L
1845	1	2	18/28	0.4465	0.4465	3.8031	UPSP:COA1_HUMAN	2	R.QVQAEVPGSPIFVMR.L
1060	1	3	21/44	0.3143	0.3143	3.7989	UPSP:COA1_HUMAN	4	R.RVDPVYIHLAER.L
1248	1	2	10/12	0.1041	0.1041	2.0821	UPSP:COA1_HUMAN	2	R.TFEDFVR.I
1508	1	3	35/72	0.4251	0.4251	4.4365	UPSP:COA1_HUMAN	4	R.TIQVENSHLILTGAGALNK.V
1438	1	2	13/18	0.1969	0.1969	2.2359	UPSP:COA1_HUMAN	4	R.YLYLTPQDYK.R
0080	1	2	17/26	0.3687	0.3687	3.3920	UPSP:P53_HUMAN	30	K.KGEPHHELPPGSTK.R
1532	1	3	29/52	0.3007	0.3007	4.9203	UPSP:P53_HUMAN	24	K.KKPLDGEYFTLQIR.G
1723	1	3	24/48	0.1675	0.1675	3.6764	UPSP:P53_HUMAN	24	K.KPLDGEYFTLQIR.G
0416	1	2	14/18	0.1857	0.1857	2.2124	UPSP:P53_HUMAN	37	K.QSQHMTEVVR.R
0366	1	2	20/26	0.2899	0.2899	3.8375	UPSP:P53_HUMAN	28	K.RALPNNTSSSPQPK.K
0767	1	1	11/16	0.2938	0.2938	2.4728	UPSP:P53_HUMAN	27	K.TYQGSYGFR.L
0404	1	2	19/24	0.5982	0.5982	2.8052	UPSP:P53_HUMAN	28	R.ALPNNTSSSPQPK.K
0058	1	1	7/10	0.0095	0.0095	1.6279	UPSP:P53_HUMAN	35	R.AM*AIYK.Q
1173	1	2	13/16	0.1569	0.1569	1.8805	UPSP:P53_HUMAN	25	R.ELNEALELK.D
0941	1	2	23/28	0.4263	0.4263	5.0268	UPSP:P53_HUMAN	25	R.ELNEALELKDAQAGK.E
0859	1	3	35/80	0.4367	0.4367	5.4777	UPSP:P53_HUMAN	25	R.ELNEALELKDAQAGKEPGGSR.
0651	1	2	10/12	0.1615	0.1615	1.6149	UPSP:P53_HUMAN	25	R.ERFEM*FR.E
0011	1	3	23/56	0.2887	0.2887	3.3791	UPSP:P53_HUMAN	30	R.KKGEPHHELPPGSTK.R
0749	1	2	17/18	0.1872	0.1872	3.0995	UPSP:P53_HUMAN	26	R.LGFLHSGTAK.S
0515	1	1	7/10	0.0158	0.0158	1.8705	UPSP:P53_HUMAN	28	R.NSFEVR.V
2288	1	3	35/72	0.1024	0.1024	5.7571	UPSP:P53_HUMAN	27	R.RPILTIITLEDSSGNLLGR.N

0081	1	2	12/14	0.0540	0.0540	2.9933	UPSP:P53_HUMAN	30	R.RTEEENLR.K
0004	1	2	11/12	0.0026	0.0026	2.3992	UPSP:P53_HUMAN	30	R.TEEENLR.K
1140	1	3	26/48	0.3702	0.3702	3.5180	UPSP:P53_HUMAN	36	R.VEGNLRVEYLDDR.N
0650	1	2	11/12	0.2360	0.2360	2.5555	UPSP:P53_HUMAN	36	R.VEYLDDR.N
1062	1	2	17/22	0.2795	0.2795	2.9427	UPSP:BRE1A_HUMAN	4	K.AM*EAAQLADDLK.A
1232	1	2	21/24	0.3995	0.3995	3.9278	UPSP:BRE1A_HUMAN	8	K.LGGVSSTEELDIR.T
1537	1	3	36/64	0.3368	0.3368	5.6092	UPSP:BRE1A_HUMAN	4	K.LLKEEKEELADQVLTLK.T
2166	1	2	12/28	0.2069	0.2069	3.0108	UPSP:BRE1A_HUMAN	5	K.RYDLEQGLGDLLTER.K
1085	1	2	14/20	0.2262	0.2262	2.9908	UPSP:BRE1A_HUMAN	4	R.AVEEQIEYLQK.K
2464	1	3	27/120	0.3548	0.3548	3.7767	UPSP:BRE1A_HUMAN	4	R.ERGEGQEPAFSFLATLASSSSEE
2597	1	3	45/112	0.5618	0.5618	6.6256	UPSP:BRE1A_HUMAN	4	R.GEGQEPAFSFLATLASSSSEEM
0630	1	2	13/14	0.2173	0.2173	2.6394	UPSP:BRE1A_HUMAN	8	R.HLAEVLER.V
1485	1	3	28/68	0.3131	0.3131	3.6159	UPSP:BRE1A_HUMAN	8	R.IEFEQTLAANEQAGPINR.E
1815	1	2	17/20	0.2566	0.2566	3.5821	UPSP:BRE1A_HUMAN	9	R.LQELTDLLQEK.H
1350	1	2	19/24	0.3494	0.3494	3.5926	UPSP:BRE1A_HUMAN	8	R.QATDDASLLIVNR.Y
0882	1	2	16/22	0.4176	0.4176	3.0576	UPSP:BRE1A_HUMAN	8	R.RAVSQIVTVYDK.L
1973	1	2	20/26	0.2872	0.2872	4.0944	UPSP:BRE1A_HUMAN	15	R.TEVIQLEDTLAQVR.K
1915	1	2	21/24	0.4845	0.4845	4.2966	UPSP:MCCC2_HUMAN	_	K.AFYGDTLVTGFAR.I
0871	1	2	13/18	0.1919	0.1919	1.8621	UPSP:MCCC2_HUMAN	1	K.GGAYYPVTVK.K
2666	1	3	39/108	0.4376	0.4376	6.1130	UPSP:MCCC2_HUMAN	1	K.KLDVTIEPSEEPLFPADELYGIV
1291	1	3	23/60	0.2076	0.2076	3.0954	UPSP:MCCC2_HUMAN	1	K.QFSSADEAALKEPIIK.K
1907	1	2	15/24	0.3516	0.3516	2.4180	UPSP:MCCC2_HUMAN	_	K.QGTIFLAGPPLVK.A
2013	1	2	13/16	0.2349	0.2349	2.3007	UPSP:MCCC2_HUMAN	_	R.FLYIWPNAR.I
2364	1	2	23/40	0.5978	0.5978	4.7750	UPSP:MCCC2_HUMAN	_	R.IFGYPVGIVGNNGVLFSESAK.K
1964	1	2	26/34	0.4930	0.4930	5.1635	UPSP:MCCC2_HUMAN	1	R.ISVM*GGEQAANVLATITK.D
2241	1	2	23/34	0.4602	0.4602	5.6636	UPSP:MCCC2_HUMAN	1	R.ISVMGGEQAANVLATITK.D
2811	1	2	26/32	0.5329	0.5329	5.5756	UPSP:MCCC2_HUMAN	_	R.NIPLLFLQNITGFM*VGR.E
1530	1	2	14/24	0.2455	0.2455	2.6167	UPSP:MCCC2_HUMAN	1	R.VWDDGIIDPADTR.L
1141	1	2	13/18	0.0676	0.0676	2.4608	UPSP:NUCL_HUMAN	5	K.EVFEDAAEIR.L
1871	1	2	20/26	0.4093	0.4093	4.0127	UPSP:NUCL_HUMAN	6	K.FGYVDFESAEDLEK.A
1809	1	2	20/26	0.4506	0.4506	4.2090	UPSP:NUCL_HUMAN	7	K.GFGFVDFNSEEDAK.A
1507	1	2	13/14	0.2464	0.2464	2.0899	UPSP:NUCL_HUMAN	6	K.GIAYIEFK.T
1024	1	2	15/16	0.2965	0.2965	2.8115	UPSP:NUCL_HUMAN	5	K.NDLAVVDVR.I
2446	1	3	24/88	0.0669	0.0669	3.5139	UPSP:NUCL_HUMAN	5	K.QKVEGTEPTTAFNLFVGNLNF
0552	1	2	14/22	0.0998	0.0998	1.9971	UPSP:NUCL_HUMAN	6	K.TEADAEKTFEEK.Q
2678	1	2	25/42	0.5219	0.5219	5.6765	UPSP:NUCL_HUMAN	6	K.TLVLSNLSYSATEETLQEVFEK
2590	1	3	37/80	0.4432	0.4432	5.2581	UPSP:NUCL_HUMAN	5	K.VEGTEPTTAFNLFVGNLNFNK.
0012	1	2	10/12	0.0344	0.0344	2.2444	UPSP:NUCL_HUMAN	5	K.VTQDELK.E
2203	1	2	24/32	0.4983	0.4983	5.2701	UPSP:NUCL_HUMAN	5	K.VTQDELKEVFEDAAEIR.L
2185	1	2	15/32	0.0326	0.0326	2.2781	UPSP:MCCA_HUMAN	3	K.AVNYVGAGTVEFIMDSK.H
1778	1	2	17/22	0.4161	0.4161	3.0950	UPSP:MCCA_HUMAN	3	K.EGSIEIDIPVPK.Y
1582	1	3	35/68	0.4293	0.4293	5.6972	UPSP:MCCA_HUMAN	3	K.IPLSQEEITLQGHAFEAR.I
2005	1	2	19/28	0.4779	0.4779	4.0080	UPSP:MCCA_HUMAN	2	K.QEGIIFIGPPPSAIR.D
1222	1	3	25/92	0.0834	0.0834	3.1052	UPSP:MCCA_HUMAN	3	K.YLSSVSSQETQGGPLAPM*TGT
1820	1	3	39/92	0.4120	0.4120	5.6624	UPSP:MCCA_HUMAN	3	R.IYAEDPSNNFM*PVAGPLVHLS
2156	1	3	33/92	0.3192	0.3192	4.5049	UPSP:MCCA_HUMAN	3	R.IYAEDPSNNFMPVAGPLVHLST
1113	1	2	20/24	0.4160	0.4160	4.9136	UPSP:MCCA_HUMAN	2	R.SEQEFQEQLESAR.R
0620	1	2	19/30	0.1118	0.1118	2.5035	UPSP:BRE1B_HUMAN	6	K.EM*APVPGTTTTTTSVK.K
1444	1	3	28/64	0.2667	0.2667	5.2207	UPSP:BRE1B_HUMAN	8	K.LLREEKDELGEQVLGLK.S
1115	1	2	18/22	0.3676	0.3676	3.6151	UPSP:BRE1B_HUMAN	7	K.SQVDAQLLTVQK.L
1952	1	2	21/32	0.4560	0.4560	4.6472	UPSP:BRE1B_HUMAN	4	K.VLEM*ETTVEDLQWDIEK.L
1521	1	2	22/30	0.3645	0.3645	3.4267	UPSP:BRE1B_HUMAN	6	R.EGPSLGPPPVASALSR.A
1267	1	2	18/20	0.3533	0.3533	2.7819	UPSP:BRE1B_HUMAN	4	R.ISLEYSELQDK.V
2087	1	3	33/76	0.3692	0.3692	5.3779	UPSP:BRE1B_HUMAN	8	R.KVEVYADADEILQEEIKEYK.A
1088	1	2	15/20	0.1502	0.1502	2.6961	UPSP:BRE1B_HUMAN	4	R.LQDLATQLQEK.H
1382	1	2	16/18	0.4131	0.4131	2.9936	UPSP:FBXW8_HUMAN	1	K.ILVYSLEAGR.R
2001	1	2	19/30	0.4872	0.4872	4.6015	UPSP:FBXW8_HUMAN	1	K.IVSGGEEGLVSVWDYR.M
1799	1	2	17/22	0.2902	0.2902	2.9846	UPSP:FBXW8_HUMAN	1	K.LVQYLEIVPETR.R
1784	1	2	18/20	0.3156	0.3156	3.7356	UPSP:FBXW8_HUMAN	5	K.VIAEDEVLWYR.L
1804	1	2	17/28	0.1437	0.1437	2.4311	UPSP:FBXW8_HUMAN	_	R.EGAGGGEQLVDQLIR.D
0792	1	2	17/20	0.2969	0.2969	3.3667	UPSP:FBXW8_HUMAN	1	R.NADLDSFTTHR.R
1264	1	3	24/52	0.3944	0.3944	3.7549	UPSP:FBXW8_HUMAN	1	R.SGNIALSLSAHQLR.V
2110	1	2	21/28	0.4034	0.4034	3.8415	UPSP:PYC_HUMAN	2	K.DFTATFGPLDSLNTR.L
2259	1	2	19/30	0.2481	0.2481	3.6103	UPSP:PYC_HUMAN	2	K.IVGDLAQFMVQNGLSR.A
2562	1	2	16/24	0.3410	0.3410	2.4562	UPSP:PYC_HUMAN	1	R.ELIPNIPFQM*LLR.G
2375	1	3	43/92	0.4751	0.4751	6.6618	UPSP:PYC_HUMAN	2	R.HGEEVTPEDVLSAAM*YPDVF

2606	1	3	29/84	0.3943	0.3943	3.6557	UPSP:PYC_HUMAN	2	R.SVVEFLQGYIGVPHGGFPEPFR.
1763	1	2	16/22	0.2812	0.2812	2.9399	UPSP:PYC_HUMAN	2	R.VFDYSEYWEGAR.G
2356	1	3	32/84	0.3667	0.3667	5.3157	UPSP:HNRPU_HUMAN	9	K.EKPYFPIPEEYTFIQNVPLEDR.
1883	1	2	20/34	0.3286	0.3286	2.5655	UPSP:HNRPU_HUMAN	5	K.SSGPTSLFAVTVAPPGAR.Q
1300	1	2	18/22	0.3521	0.3521	3.5264	UPSP:HNRPU_HUMAN	9	K.YNILGTNTIM*DK.M
1467	1	2	12/18	0.1169	0.1169	1.9407	UPSP:HNRPU_HUMAN	7	R.GYFEYIEENK.Y
1400	1	2	19/24	0.3944	0.3944	3.7037	UPSP:HNRPU_HUMAN	7	R.GYFEYIEENKYSR.A
1407	1	2	24/28	0.4174	0.4174	4.2895	UPSP:HNRPU_HUMAN	9	R.NFILDQTNVSAAAQR.R
0674	1	2	11/14	0.1399	0.1399	1.9509	UPSP:RS2_HUMAN	7	K.ATFDAISK.T
0799	1	2	12/14	0.1176	0.1176	2.0209	UPSP:RS2_HUMAN	6	K.LSIVPVRR.G
2612	1	2	19/24	0.3807	0.3807	4.5371	UPSP:RS2_HUMAN	7	K.SLEEIYLFSLPIK.E
1447	1	3	26/44	0.3816	0.3816	3.2863	UPSP:RS2_HUMAN	7	K.SPYQEFTDHLVK.T
1975	1	2	15/20	0.4989	0.4989	3.0697	UPSP:RS2_HUMAN	8	K.TYSYLTPDLWK.E
0786	1	2	13/20	0.2714	0.2714	2.4887	UPSP:RS2_HUMAN	5	R.GTGIVSAPVPK.K
1154	1	3	37/68	0.4450	0.4450	5.9912	UPTR:Q59FY4_HUMAN	3	K.IHNANPELTDGQIQAM*LR.R
2038	1	2	21/30	0.4453	0.4453	4.7449	UPTR:Q59FY4_HUMAN	3	K.IIQQAGQVWFPDSAFK.T
1752	1	2	19/22	0.3141	0.3141	4.1260	UPTR:Q59FY4_HUMAN	3	R.DIIVIGNDITYR.I
0781	1	2	18/20	0.2929	0.2929	2.6776	UPTR:Q59FY4_HUMAN	3	R.LGTPELSTAER.K
1695	1	2	20/34	0.2848	0.2848	3.3640	UPTR:Q59FY4_HUMAN	3	R.TVELSIPADPANLDSEAK.I
1653	1	2	17/22	0.2778	0.2778	3.2409	UPSP:HSP7C_HUMAN	4	K.DAGTIAGLNVLR.I
0980	1	2	22/28	0.3766	0.3766	4.5899	UPSP:HSP7C_HUMAN	3	K.NQVAM*NPTNTVFDAK.R
1989	1	3	35/92	0.5067	0.5067	5.4118	UPSP:HSP7C_HUMAN	5	K.QTQTFTTYSDNQPGVLIQVYE
1618	1	2	22/26	0.4121	0.4121	3.8951	UPSP:HSP7C_HUMAN	4	K.SFYPEEVSSM*VLTK.M
1692	1	2	15/34	0.3500	0.3500	2.9987	UPSP:HSP7C_HUMAN	4	K.TVTNAVVTVPAYFNDSQR.Q
1707	1	2	16/18	0.3086	0.3086	3.8285	UPSP:RLA0_HUMAN	6	K.IIQLLDDYPK.C
2244	1	2	20/22	0.4415	0.4415	4.1890	UPSP:RLA0_HUMAN	5	K.TSFFQALGITTK.I
1341	1	2	14/16	0.3370	0.3370	2.6663	UPSP:RLA0_HUMAN	6	R.GNVGFVFTK.E
2234	1	2	16/24	0.2969	0.2969	3.8690	UPSP:RLA0_HUMAN	4	R.GTIEILSDVQLIK.T
2236	1	2	22/32	0.5419	0.5419	5.0053	UPSP:RLA0_HUMAN	7	R.VLALSVETDYTFPLAEK.V
1874	1	3	28/68	0.2854	0.2854	3.6379	UPSP:DECR_HUMAN	_	K.FDGGEEVLISGEFNDLRK.V
2570	1	2	22/50	0.3600	0.3600	4.2523	UPSP:DECR_HUMAN	1	K.GAAFLSITTIYAETGSGFVVPS
1253	1	2	20/24	0.2538	0.2538	3.5689	UPSP:DECR_HUMAN	2	K.VAFITGGGTGLGK.G
1499	1	3	32/84	0.4068	0.4068	5.1742	UPSP:DECR_HUMAN	2	K.VAGHPNIVINNAAGNFISPTER.
1326	1	2	14/18	0.0860	0.0860	2.6367	UPSP:DECR_HUMAN	1	R.FNVIQPGPIK.T
1156	1	2	16/20	0.1874	0.1874	3.0881	UPSP:RL7A_HUMAN	4	K.NFGIGQDIQPK.R
1953	1	2	21/26	0.4256	0.4256	4.2846	UPSP:RL7A_HUMAN	4	K.VPPAINQFTQALDR.Q
1249	1	2	18/24	0.4388	0.4388	4.1237	UPSP:RL7A_HUMAN	6	R.AGVNTVTTLVENK.K
1021	1	3	23/52	0.3801	0.3801	3.6706	UPSP:RL7A_HUMAN	6	R.AGVNTVTTLVENKK.A
2086	1	3	24/60	0.1538	0.1538	3.0081	UPSP:RL7A_HUMAN	4	R.LKVPPAINQFTQALDR.Q
0519	1	2	14/16	0.2606	0.2606	2.6065	UPSP:NPM_HUMAN	18	K.GPSSVEDIK.A
2471	1	2	26/40	0.6455	0.6455	5.7557	UPSP:NPM_HUMAN	20	K.M*SVQPTVSLGGFEITPPVVLR.
2262	1	2	20/26	0.3127	0.3127	4.0045	UPSP:NPM_HUMAN	11	R.M*TDQEAIQDLWQWR.K
2048	1	3	28/108	0.3927	0.3927	4.7000	UPSP:NPM_HUMAN	18	R.TVSLGAGAKDELHIVEAEAM*
1806	1	2	22/36	0.4033	0.4033	3.9203	UPTR:Q7Z561_HUMAN	3	K.DVDDGLQAAEEVGYPVM*IK.
1938	1	3	28/88	0.3254	0.3254	3.2963	UPTR:Q7Z561_HUMAN	3	K.GYVKDVDDGLQAAEEVGYPV
2371	1	3	40/108	0.4386	0.4386	4.8472	UPTR:Q7Z561_HUMAN	3	K.M*ADHYVPVPGGPNNNNYAN
1577	1	3	25/52	0.3952	0.3952	3.3682	UPTR:Q7Z561_HUMAN	1	R.QVLIASHLPSYELR.H
0850	1	2	13/14	0.0937	0.0937	2.5458	UPTR:Q6NXR8_HUMAN	2	K.APAM*FNIR.N
0888	1	2	12/14	0.2613	0.2613	2.7062	UPTR:Q6NXR8_HUMAN	2	K.TTDGYLLR.L
2334	1	2	24/32	0.6045	0.6045	5.1723	UPTR:Q6NXR8_HUMAN	2	R.VFEVSLADLQNDEVAFR.K
2130	1	3	29/68	0.2797	0.2797	3.8576	UPTR:Q6NXR8_HUMAN	2	R.VFEVSLADLQNDEVAFRK.F
0950	1	2	13/14	0.1906	0.1906	2.6791	UPSP:RL3_HUMAN	9	K.IGQGYLIK.D
2361	1	3	31/84	0.3941	0.3941	3.8369	UPSP:RL3_HUMAN	9	K.SINPLGGFVHYGEVTNDFVM*
1939	1	3	37/100	0.4700	0.4700	4.7769	UPSP:RL3_HUMAN	10	R.ERLEQQVPVNQVFGQDEM*ID
1078	1	2	14/16	0.3419	0.3419	2.9688	UPSP:RL3_HUMAN	6	R.HGSLGFLPR.K
1371	1	2	20/24	0.3720	0.3720	3.6805	UPTR:Q53HV1_HUMAN	3	K.VNDTIQIDLETGK.I
1656	1	2	15/16	0.1896	0.1896	3.3526	UPTR:Q53HV1_HUMAN	3	R.LSNIFVIGK.G
2055	1	2	17/32	0.3737	0.3737	3.9898	UPTR:Q53HV1_HUMAN	3	R.TDITYPAGFM*DVISIDK.T
2642	1	2	27/32	0.5605	0.5605	6.1024	UPSP:PCCB_HUMAN	6	R.IQEGVESLAGYADIFLR.N
0453	1	2	14/24	0.2505	0.2505	2.2188	UPSP:PCCB_HUMAN	5	R.KAYGGAYDVM*SSK.H
2112	1	2	16/28	0.0880	0.0880	2.6168	UPSP:PCCB_HUMAN	6	R.LVPELDTIVPLESTK.A
1382	1	3	23/52	0.2885	0.2885	3.0066	UPSP:HSP71_HUMAN	6	K.HWPFQVINDGDKPK.V
1343	1	2	22/28	0.4618	0.4618	4.1652	UPSP:HSP71_HUMAN	5	K.NQVALNPQNTVFDAK.R
1805	1	2	19/30	0.4619	0.4619	3.5692	UPSP:HSP71_HUMAN	9	R.IINEPTAAAIAYGLDR.T
2682	1	2	17/28	0.3169	0.3169	2.9151	UPSP:ECHA_HUMAN	3	K.ADM*VIEAVFEDLSLK.H
2662	1	3	26/108	0.2977	0.2977	3.6093	UPSP:ECHA_HUMAN	2	K.KLDSLTTSFGFPVGAATLVDE

1770	1	2	18/28	0.4445	0.4445	3.2920	UPSP:ECHA_HUMAN	3	R.FGGGNPELLTQMVSK.G
0502	1	2	19/20	0.2434	0.2434	4.0519	UPSP:RL4_HUMAN	3	K.AAAAAAALQAK.S
1302	1	2	16/18	0.3121	0.3121	3.1547	UPSP:RL4_HUMAN	5	R.KLDELYGTWR.K
1912	1	2	19/22	0.4791	0.4791	2.5720	UPSP:RL4_HUMAN	5	R.NIPGITLLNVSK.L
1889	1	2	20/28	0.4261	0.4261	3.5512	UPSP:RS24_HUMAN	2	K.TTGFGM*IYDSLDYAK.K
2189	1	2	16/28	0.2150	0.2150	2.6695	UPSP:RS24_HUMAN	2	K.TTGFGMIYDSLDYAK.K
2579	1	2	17/22	0.2631	0.2631	2.7347	UPSP:RS24_HUMAN	2	K.TTPDVIFVFGFR.T
1231	1	2	14/22	0.0081	0.0081	2.6795	UPSP:H12_HUMAN	6	K.ASGPPVSELITK.A
0967	1	3	25/48	0.2636	0.2636	3.9644	UPSP:H12_HUMAN	6	R.KASGPPVSELITK.A
1104	1	2	15/16	0.0220	0.0220	2.2713	UPSP:H12_HUMAN	6	R.SGVSLAALK.K
2542	1	2	21/32	0.3624	0.3624	4.7810	UPSP:RL6_HUMAN	5	K.QLASGLLLVTGPLVLNR.V
2803	1	2	21/28	0.5225	0.5225	4.6157	UPSP:RL6_HUMAN	6	R.ASITPGTILIILTGR.H
2187	1	2	23/28	0.4155	0.4155	5.0489	UPSP:TBA6_HUMAN	8	R.AVFVDLEPTVIDEVR.T
2644	1	2	19/26	0.4719	0.4719	4.0429	UPSP:TBA6_HUMAN	8	R.LISQIVSSITASLR.F
1959	1	2	25/38	0.5911	0.5911	6.0315	UPSP:TBA2_HUMAN	15	K.TIGGGDDSFNTFFSETGAGK.H
1601	1	2	15/34	0.3359	0.3359	2.8232	UPSP:TBA2_HUMAN	21	K.VGINYQPPTVVPGGDLAK.V
1779	1	2	26/30	0.0255	0.0255	5.1493	UPSP:GRP78_HUMAN	18	R.IINEPTAAAIAYGLDK.R
0603	1	2	19/20	0.2454	0.2454	3.6714	UPSP:GRP78_HUMAN	26	R.VEIIANDQGNR.I
0517	1	2	21/32	0.5582	0.5582	4.7034	UPTR:Q96FW6_HUMAN	8	K.QGPVSQSATQQPVTADK.Q
1555	1	3	28/96	0.4667	0.4667	4.0531	UPTR:Q96FW6_HUMAN	8	R.ISTPQTNTVPIKPLISTPPVSSQP
1916	1	2	16/26	0.3237	0.3237	3.3117	UPSP:G3P_HUMAN	4	K.LISWYDNEFGYSNR.V
2392	1	3	33/92	0.4709	0.4709	5.1238	UPSP:G3P_HUMAN	5	K.VIHDNFGIVEGLM*TTVHAITA
1434	1	2	19/20	0.3255	0.3255	3.4854	UPSP:RL7_HUMAN	6	R.IALTDNALIAR.S
2163	1	2	20/26	0.4475	0.4475	4.2653	UPSP:RL7_HUMAN	7	R.IVEPYIAWGYPNLK.S
1098	1	2	15/20	0.3329	0.3329	2.8174	UPTR:Q9BS10_HUMAN	2	-.LTPEEEEILNK.K
1427	1	2	18/24	0.3929	0.3929	4.5049	UPTR:Q9BS10_HUMAN	2	K.ISSLLEEQFQQGK.L
0944	1	2	18/24	0.4262	0.4262	3.4422	UPTR:Q6IPX9_HUMAN	5	R.ELAEDGYSGVEVR.V
1867	1	2	22/26	0.4260	0.4260	3.7986	UPTR:Q6IPX9_HUMAN	5	R.FGFPEGSVELYAEK.V
2221	1	2	16/34	0.3061	0.3061	3.3998	UPSP:RL21_HUMAN	4	R.TNGKEPELLEPIPYEFM*A.-
1185	1	2	19/28	0.2666	0.2666	3.7074	UPSP:RL21_HUMAN	4	R.VYNVTQHAVGIVVNK.Q
1836	1	2	17/38	0.3107	0.3107	3.0159	UPSP:SYR_HUMAN	2	K.AAYPDLENPPLLVTPSQQAK.F
1376	1	2	20/30	0.3539	0.3539	3.7399	UPSP:SYR_HUMAN	3	K.SDGGYTYDTSDLAAIK.Q
1152	1	2	13/16	0.3897	0.3897	2.5476	UPTR:Q59FI9_HUMAN	2	K.IGPLGLSPK.K
1933	1	2	18/30	0.1921	0.1921	3.9486	UPTR:Q59FI9_HUMAN	1	R.QAQIEVVPSASALIIK.A
1273	1	2	17/24	0.2835	0.2835	3.1115	UPTR:Q9NQS8_HUMAN	5	K.DEILPTTPISEQK.G
0660	1	2	14/30	0.1878	0.1878	2.9595	UPTR:Q9NQS8_HUMAN	4	K.GGKPEPPAM*PQPVPTA.-
2690	1	2	16/20	0.3394	0.3394	3.2514	GP:M77233_1	1	K.DVNFEFPEFQL.-
2182	1	2	13/24	0.2226	0.2226	2.6203	GP:M77233_1	1	R.KAIIIFVPVPQLK.S
1089	1	2	12/14	0.1944	0.1944	2.4924	UPSP:RS9_HUMAN	4	K.LIGEYGLR.N
1464	1	2	13/16	0.2670	0.2670	2.8330	UPSP:RS9_HUMAN	3	R.LFEGNALLR.R
2353	1	3	40/100	0.3948	0.3948	6.7380	UPTR:Q6LC01_HUMAN	14	R.SGPFGQIFRPDNFVFGQSGAGN
2563	1	2	22/30	0.4630	0.4630	5.6777	UPSP:TCPE_HUMAN	3	K.LGFAGLVQEISFGTTK.D
2760	1	2	21/40	0.3747	0.3747	5.3964	UPSP:AP2B1_HUMAN	2	K.LAPPLVTLLSGEPEVQYVALR.
0605	1	2	25/30	0.5115	0.5115	5.3071	UPSP:MAGB2_HUMAN	2	R.APTTAAAAAAGVSSTK.S
1492	1	2	23/28	0.5035	0.5035	5.1948	UPSP:RS6_HUMAN	6	R.M*ATEVAADALGEEWK.G
1887	1	2	19/22	0.4911	0.4911	4.9522	UPSP:RL14_HUMAN	9	K.LVAIVDVIDQNR.A
2009	1	2	23/36	0.5832	0.5832	4.9048	UPSP:RL9_HUMAN	1	K.TILSNQTVDIPENVDITLK.G
1979	1	2	22/30	0.4302	0.4302	4.7423	UPTR:Q6NZ54_HUMAN	1	K.GLDVDSLVIEHIQVNK.A
2217	1	3	28/80	0.3529	0.3529	4.6446	UPTR:O75147_HUMAN	1	K.NTVLLTWKPPEPAPETPFIYR.L
2345	1	2	21/32	0.4657	0.4657	4.6122	UPSP:SDPR_HUMAN	1	K.VLIFQEENEIPASVFVK.Q
2585	1	2	21/26	0.4283	0.4283	4.5875	UPSP:LA_HUMAN	1	R.LTTDFNVIVEALSK.S
1380	1	2	22/24	0.3537	0.3537	4.5672	UPSP:RL10_HUMAN	9	K.FNADEFEDM*VAEK.R
1597	1	2	22/28	0.5120	0.5120	4.5378	UPTR:Q5JR95_HUMAN	1	R.IIDVVYNASNNELVR.T
2310	1	3	35/84	0.3552	0.3552	4.4775	UPSP:RS17_HUMAN	2	K.LLDFGSLSNLQVTQPTVGM*N
2508	1	2	19/30	0.3878	0.3878	4.4047	UPTR:Q6ICQ4_HUMAN	2	K.AAGVNVEPFWPGLFAK.A
2342	1	3	35/72	0.3121	0.3121	4.4015	GP:X60020_1		R.RPILTDITLEDSSGNLLGR.N
2272	1	2	19/24	0.4065	0.4065	4.3194	UPSP:MDM2_HUMAN	9	K.EVLFYLGQYIM*TK.R
1163	1	2	17/24	0.2584	0.2584	4.3006	UPSP:COA2_HUMAN	9	K.EASFEYLQNEGER.L
2628	1	3	32/84	0.3700	0.3700	4.2698	UPTR:Q59FS3_HUMAN	2	R.TLTAVHDAILEDLVFPSEIVGK.
1439	1	2	24/32	0.5105	0.5105	4.2348	UPTR:Q53G49_HUMAN	5	K.VWLDPNETNEIANANSR.Q
2173	1	3	37/96	0.4521	0.4521	4.1664	UPSP:IF2G_HUMAN	4	K.IVSLFAEHNDLQYAAPGGLIGV
2777	1	2	21/40	0.5114	0.5114	4.0867	UPSP:DDX1_HUMAN	1	R.FLVLDEADGLLSQGYSDFINR.
1484	1	2	21/32	0.5016	0.5016	4.0822	UPSP:DBPA_HUMAN	13	R.SVGDGETVEFDVVEGEK.G
2148	1	2	19/28	0.4273	0.4273	4.0536	UPSP:H2B1B_HUMAN	33	K.AM*GIM*NSFVNDIFER.I
1980	1	2	21/28	0.5520	0.5520	4.0073	UPTR:Q6PB27_HUMAN	1	K.HSGNITFDEIVNIAR.Q
2051	1	2	20/30	0.4046	0.4046	3.9981	UPSP:TCPD_HUMAN	3	R.ALIAGGGAPEIELALR.L

1426	1	3	31/76	0.3553	0.3553	3.9849	UPSP:SYD_HUMAN	6	R.LPLQLDDAVRPEAEGEEEGR.A
2252	1	2	18/26	0.3502	0.3502	3.8803	UPTR:Q6IRZ0_HUMAN	1	K.LYTLVTYVPVTTFK.S
0665	1	2	18/30	0.4131	0.4131	3.8791	UPSP:HSP72_HUMAN	8	K.STAGDTHLGGEDFDNR.M
1881	1	3	30/92	0.1680	0.1680	3.8397	UPTR:Q5VTE0_HUMAN	22	R.VETGVLKPGM*VVTFAPVNVT
1354	1	2	18/22	0.4434	0.4434	3.7650	UPSP:KC1AL_HUMAN	8	K.TVLM*LADQM*ISR.I
0603	1	2	19/20	0.2454	0.2454	3.6714	UPSP:HSP76_HUMAN	3	R.VEILANDQGNR.T
1662	1	2	18/24	0.3336	0.3336	3.6204	UPTR:Q1JQ76_HUMAN	4	K.KYDAFLASESLIK.Q
2634	1	2	20/24	0.2686	0.2686	3.5966	UPSP:HNRPQ_HUMAN	10	R.DLFEDELVPLFEK.A
2205	1	2	20/28	0.4041	0.4041	3.5878	UPTR:Q5JP53_HUMAN	5	R.ALTVPELTQQVFDAK.N
1287	1	2	19/24	0.3500	0.3500	3.5772	GP:AE006463_8	3	R.LAPDYDALDVANK.I
1609	1	2	18/26	0.2954	0.2954	3.5572	UPSP:HS90A_HUMAN	20	R.GVVDSEDLPLNISR.E
1773	1	2	18/30	0.3901	0.3901	3.5001	UPSP:MDM4_HUMAN	3	R.EDEDLIENLAQDETSR.L
1227	1	2	18/24	0.3775	0.3775	3.4994	UPSP:HS70L_HUMAN	27	R.TTPSYVAFTDTER.L
1358	1	2	18/20	0.4155	0.4155	3.4970	UPSP:PCCA_HUMAN	4	R.M*ADALDNYVIR.G
0385	1	2	18/28	0.4106	0.4106	3.4779	UPSP:TCPB_HUMAN	4	R.AAHSEGNTTAGLDM*R.E
2049	1	3	20/80	0.1471	0.1471	3.3624	UPSP:HNRPD_HUMAN	8	K.IREYFGGFGEVESIELPM*DNK.
1475	1	2	19/26	0.4819	0.4819	3.3607	UPTR:Q5VVC8_HUMAN	8	K.VLEQLTGQTPVFSK.A
1678	1	2	17/36	0.3792	0.3792	3.3566	UPTR:Q53EN9_HUMAN	6	K.HSSDASSLLPQNILSQTSR.H
2042	1	2	15/24	0.3959	0.3959	3.3510	UPSP:RL18_HUMAN	3	K.ILTFDQLALDSPK.G
1808	1	3	26/84	0.2092	0.2092	3.1020	UPSP:SYK_HUMAN	2	K.VTYHPDGPEGQAYDVDFTPPFI
2054	1	2	16/38	0.1795	0.1795	3.1013	UPTR:Q5JWB0_HUMAN	_	R.QPMEPPMEPSGGEQEPGAVR.F
1083	1	2	16/24	0.3550	0.3550	3.0771	UPSP:RL13_HUMAN	5	K.LATQLTGPVM*PVR.N
1251	1	2	18/24	0.4349	0.4349	3.0693	GP:AY891881_1	_	R.AITGASLADIM*AK.R
1736	1	2	19/30	0.3051	0.3051	2.8143	UPSP:DHX9_HUMAN	3	R.ELDALDANDELTPLGR.I

Supplementary Table S2. Affinaty purification and mass spectrometry analysis of p53 in K562 cell.

GYGI LAB / TAPLIN BIOLOGICAL MASS SPEC FACILITY SearchWiz | Status | Browse

/home/www/production/html/saved_sets/13234.2.sumlist

226 peptides, **44** proteins Charge states: 1+ ☑ 2+ ☑ 3+ ☑ 4+ ☑ Sort: Reference desc ◉ html ○ text ○ dta Peptides Reload

13234.2 Lookup ○ peptide ◉ reference ☐ Group 2/3+ ☐ Use best ion

scanf, scanl, Rank, Rank_Sp, charge, Ions, premass, _M_H_, mass_diff

R: ☐ H: ☐ Sequence:
R: ☐ H: ☐ Peptide type: tryptic
R: ☐ H: ☐ AA type: acidic
R: ☐ H: ☐ Mod AA type: acidic

XCorr 1+ 0; XCorr 2+ 0; XCorr 3+ 0; XCorr 4+ 0; dCn: ; dCn2:

Rank (1 to n): ; Low PPM1: ; High PPM1: ; Low CalPPM: ; High CalPPM: ; Vista Score:

☐ Hide duplicate peptides

Protein matches					☐	scanf	Rank	charge	Ions	dCn	dCn2	XCorr	Reference	Redu	Peptide
**52**	**52**	3.473	0.00	UPSP:CUL7_HUMAN	☐	0723	1	2	15/18	0.3019	0.3019	2.8911	UPSP:CUL7_HUMAN	2	K.AAHVSEQFAR.H
**21**	**21**	3.853	0.00	UPSP:PARC_HUMAN	☐	0827	1	2	15/24	0.3441	0.3441	1.9936	UPSP:CUL7_HUMAN	2	K.EPPSQSPNTPLQR.L
**17**	**17**	3.873	0.00	UPSP:COA1_HUMAN	☐	2350	1	2	26/34	0.5922	0.5922	6.1007	UPSP:CUL7_HUMAN	2	K.ILLDLEQALSSEGTQENK.V
**14**	**14**	3.682	0.00	UPSP:BRE1A_HUMAN	☐	1512	1	2	28/46	0.5441	0.5441	5.9527	UPSP:CUL7_HUMAN	2	K.M*LGEDGQVIGPSQESAGEVG/
**13**	**13**	3.239	0.00	UPSP:P53_HUMAN	☐	1681	1	2	28/46	0.4561	0.4561	4.6185	UPSP:CUL7_HUMAN	2	K.MLGEDGQVIGPSQESAGEVGA
**11**	**11**	3.686	0.00	UPSP:BRE1B_HUMAN	☐	1935	1	2	17/20	0.3613	0.3613	3.3547	UPSP:CUL7_HUMAN	2	K.QVNNFLTSSWR.D
**10**	**10**	3.968	0.00	UPSP:MCCA_HUMAN	☐	2247	1	2	10/34	0.1638	0.1638	2.7660	UPSP:CUL7_HUMAN	2	K.QVNNFLTSSWRDDDFVPR.Y
**9**	**9**	4.036	0.00	UPSP:MCCC2_HUMAN	☐	0547	1	2	12/16	0.1432	0.1432	2.7252	UPSP:CUL7_HUMAN	2	K.SQSHPALER.G
**9**	**9**	3.278	0.00	UPSP:PYC_HUMAN	☐	0963	1	2	18/20	0.4060	0.4060	3.8740	UPSP:CUL7_HUMAN	2	K.SVLEEM*ETDVK.S
**7**	**7**	3.514	0.00	UPSP:FBXW8_HUMAN	☐	0498	1	2	13/16	0.2698	0.2698	2.3799	UPSP:CUL7_HUMAN	8	K.VEVSSNPHR.A
**4**	**4**	4.437	0.00	UPSP:PCCB_HUMAN	☐	1638	1	3	22/36	0.1779	0.1779	3.6968	UPSP:CUL7_HUMAN	2	K.VKPLLLQLQR.Q
**4**	**4**	4.312	0.00	UPSP:HSP7C_HUMAN	☐	0606	1	2	10/14	0.0878	0.0878	2.0723	UPSP:CUL7_HUMAN	2	R.DTLQPGM*R.V
**4**	**4**	3.568	0.00	UPSP:PCCA_HUMAN	☐	1899	1	2	18/22	0.3753	0.3753	3.9073	UPSP:CUL7_HUMAN	2	R.DYAVVLNQLGAR.D
**4**	**4**	2.941	0.00	UPSP:HS70L_HUMAN	☐	2856	1	2	18/22	0.2091	0.2091	4.1899	UPSP:CUL7_HUMAN	2	R.EVLQELIFFLHR.L
**3**	**3**	5.016	0.00	UPSP:NUCL_HUMAN	☐	1532	1	2	22/24	0.3222	0.3222	2.9642	UPSP:CUL7_HUMAN	8	R.FLPDDEAAQALGK.T
**3**	**3**	3.879	0.00	UPSP:RLA0_HUMAN	☐	2502	1	2	12/14	0.0155	0.0155	2.4940	UPSP:CUL7_HUMAN	7	R.FWPIIQIR.I
**3**	**3**	3.713	0.00	UPTR:Q59FY4_HUMAN	☐	2693	1	2	21/30	0.4005	0.4005	4.0302	UPSP:CUL7_HUMAN	2	R.GLEILGPKPTFWPVFR.E
**3**	**3**	3.653	0.00	UPSP:NPM_HUMAN	☐	1263	1	2	10/12	0.2178	0.2178	2.3655	UPSP:CUL7_HUMAN	2	R.GPAFFSR.V
**3**	**3**	3.626	0.00	UPSP:DECR_HUMAN	☐	0854	1	2	14/16	0.3603	0.3603	3.0840	UPSP:CUL7_HUMAN	2	R.GPLDLHEQK.D
**3**	**3**	3.493	0.00	GP:M66870_1	☐	2940	1	3	34/88	0.4889	0.4889	5.1395	UPSP:CUL7_HUMAN	2	R.GQLELEFSM*AM*GTLISELVQ.
**3**	**3**	3.321	0.00	UPTR:Q53EN9_HUMAN	☐	0584	1	2	16/18	0.1951	0.1951	3.5296	UPSP:CUL7_HUMAN	2	R.HIDQQIQGSR.I
**2**	**2**	4.001	0.00	UPSP:TCPE_HUMAN	☐	0475	1	2	15/16	0.1562	0.1562	2.7356	UPSP:CUL7_HUMAN	2	R.IGGAQEM*ER.L
**2**	**2**	3.916	0.00	UPSP:HSP72_HUMAN	☐	1747	1	2	22/38	0.4079	0.4079	2.5795	UPSP:CUL7_HUMAN	2	R.LIPPQTYLQAEGEDGQNLEK.R
**2**	**2**	3.394	0.00	UPSP:RS2_HUMAN	☐	0751	1	1	9/12	0.1139	0.1139	2.1193	UPSP:CUL7_HUMAN	2	R.LNDSALR.D
**1**	**1**	5.334	0.00	UPSP:HNRPU_HUMAN	☐	1128	1	2	18/24	0.3925	0.3925	3.8407	UPSP:CUL7_HUMAN	2	R.LPQQM*LQSLSTSK.E
**1**	**1**	5.156	0.00	UPSP:GRP78_HUMAN	☐	1671	1	2	20/24	0.3200	0.3200	3.9312	UPSP:CUL7_HUMAN	2	R.LPQQMLQSLSTSK.E
**1**	**1**	4.629	0.00	UPSP:TBA6_HUMAN	☐	0891	1	2	15/18	0.1122	0.1122	2.3226	UPSP:CUL7_HUMAN	2	R.LVEGYGPAGK.I
**1**	**1**	4.503	0.00	UPSP:RL4_HUMAN	☐	0759	1	2	19/24	0.3601	0.3601	3.8854	UPSP:CUL7_HUMAN	2	R.M*IQALSSHDAGTR.T
**1**	**1**	4.364	0.00	UPTR:Q96FW6_HUMAN	☐	3066	1	2	12/32	0.0314	0.0314	1.6230	UPSP:CUL7_HUMAN	8	R.M*LDDYEEISAGDEGEFR.Q
**1**	**1**	4.256	0.00	UPSP:COA2_HUMAN	☐	0868	1	2	20/24	0.3584	0.3584	3.7465	UPSP:CUL7_HUMAN	2	R.MIQALSSHDAGTR.T
**1**	**1**	4.236	0.00	UPSP:HSP71_HUMAN	☐	0639	1	2	11/14	0.2300	0.2300	1.9013	UPSP:CUL7_HUMAN	2	R.QEQNFADR.F
**1**	**1**	4.035	0.00	GP:BC022805_1	☐	2351	1	2	24/30	0.4581	0.4581	4.7000	UPSP:CUL7_HUMAN	2	R.QFHVYQLQQLDQELLK.L
**1**	**1**	4.032	0.00	UPTR:Q9NQS8_HUMAN	☐	1869	1	2	23/32	0.3992	0.3992	5.2884	UPSP:CUL7_HUMAN	7	R.QLTLLVASEDSSYM*PAR.V
**1**	**1**	4.032	0.00	UPSP:RL9_HUMAN	☐	2101	1	2	21/32	0.4481	0.4481	3.2710	UPSP:CUL7_HUMAN	7	R.QLTLLVASEDSSYMPAR.V
**1**	**1**	3.983	0.00	UPTR:Q5VVC8_HUMAN	☐	2307	1	2	16/36	0.5082	0.5082	3.5045	UPSP:CUL7_HUMAN	2	R.QPQPFLALM*QSLDTPETNR.T
**1**	**1**	3.961	0.00	UPSP:TCPH_HUMAN	☐	2819	1	2	17/36	0.4491	0.4491	3.5926	UPSP:CUL7_HUMAN	2	R.QPQPFLALMQSLDTPETNR.T
**1**	**1**	3.855	0.00	UPSP:IF2B_HUMAN	☐	2251	1	2	15/34	0.3576	0.3576	2.8875	UPSP:CUL7_HUMAN	2	R.QSNNGVPPVQVFWESTGR.T
**1**	**1**	3.853	0.00	UPSP:RL7_HUMAN	☐	1712	1	2	19/26	0.4180	0.4180	4.1807	UPSP:CUL7_HUMAN	2	R.SEFASGNTYALYVR.D
**1**	**1**	3.838	0.00	UPSP:MUC2_HUMAN	☐	1049	1	2	12/14	0.1570	0.1570	2.2688	UPSP:CUL7_HUMAN	2	R.SLLHLSSR.L
**1**	**1**	3.791	0.00	UPTR:Q7Z561_HUMAN	☐	2315	1	3	34/104	0.3650	0.3650	4.1854	UPSP:CUL7_HUMAN	2	R.SPGSIFQPQLADVSPGLPAAQA(
**1**	**1**	3.478	0.00	UPSP:EFG2_HUMAN	☐	2761	1	3	30/56	0.3340	0.3340	4.2930	UPSP:CUL7_HUMAN	1	R.THRPINIPFFDVFLR.Y
**1**	**1**	3.458	0.00	UPSP:G3PT_HUMAN	☐	1337	1	2	11/14	0.0594	0.0594	1.5181	UPSP:CUL7_HUMAN	3	R.TLHLTVLR.I
**1**	**1**	3.339	0.00	UPTR:Q8TCX0_HUMAN	☐	1911	1	2	22/28	0.2594	0.2594	4.4532	UPSP:CUL7_HUMAN	2	R.TQILLSLSQQEAIEK.H
**1**	**1**	3.112	0.00	UPSP:RL18_HUMAN	☐	0727	1	2	16/22	0.3536	0.3536	3.1154	UPSP:CUL7_HUMAN	2	R.VGHDGHPEYQIR.W
						1953	1	2	15/16	0.2437	0.2437	3.3498	UPSP:CUL7_HUMAN	2	R.VILLENLTR.F
						2026	1	2	19/32	0.4069	0.4069	3.4539	UPSP:CUL7_HUMAN	2	R.VLDLLM*HM*LSSPDYQIR.W
						2620	1	3	29/64	0.0758	0.1154	3.4372	UPSP:CUL7_HUMAN	2	R.VLDLLM*HMLSSPDYQIR.W
						2347	1	2	17/32	0.2741	0.4635	3.7826	UPSP:CUL7_HUMAN	2	R.VLDLLMHM*LSSPDYQIR.W
						1865	1	2	11/12	0.0605	0.0605	2.2046	UPSP:CUL7_HUMAN	2	R.VLFSLVK.R
						1850	1	2	22/30	0.5251	0.5251	5.2466	UPSP:CUL7_HUMAN	2	R.VPLGPGLHAYPDELIR.Q
						0627	1	2	14/16	0.0504	0.0504	2.1469	UPSP:CUL7_HUMAN	2	R.WDQASDRPR.S
						2262	1	3	48/128	0.5602	0.5602	6.9118	UPSP:CUL7_HUMAN	2	R.YLHVTSLLDQLNDSAAEPGAQ]

2100	1	2	22/26	0.3391	0.3391	4.8429	UPSP:PARC_HUMAN	6	K.FNQTEEVSVETLLK.D
1974	1	2	25/32	0.2676	0.2676	5.3298	UPSP:PARC_HUMAN	5	K.M*LAVASSSEIPTFVTGR.D
1819	1	2	22/24	0.4037	0.4037	4.9924	UPSP:PARC_HUMAN	4	K.M*LVELLTNQVGEK.M
2187	1	2	13/14	0.1602	0.1602	2.8758	UPSP:PARC_HUMAN	5	K.TLLLSVLR.V
2214	1	2	24/38	0.5601	0.5601	4.9680	UPSP:PARC_HUMAN	5	R.AALETPIIQGQDGSPELLIR.S
1172	1	2	14/16	0.2963	0.2963	3.3185	UPSP:PARC_HUMAN	5	R.AGSELFGPR.A
2334	1	2	23/32	0.5442	0.5442	4.7618	UPSP:PARC_HUMAN	6	R.FEGSTLNDLLNSQIYTK.Y
2661	1	2	11/30	0.1313	0.1313	2.5466	UPSP:PARC_HUMAN	5	R.GVEVLGPKPTFWPLFR.E
1882	1	3	24/48	0.3203	0.3203	3.5379	UPSP:PARC_HUMAN	6	R.LLAILQHSAQDFR.V
1687	1	2	15/18	0.3530	0.3530	2.6559	UPSP:PARC_HUMAN	5	R.LQAYPEELIR.Q
2711	1	2	20/24	0.3291	0.3291	5.0947	UPSP:PARC_HUMAN	5	R.LQQETQPFLLLLR.T
1809	1	2	12/16	0.0361	0.0361	1.6973	UPSP:PARC_HUMAN	6	R.QFHLFQLQR.L
2333	1	2	14/34	0.3397	0.3397	2.9030	UPSP:PARC_HUMAN	4	R.QSNNGIPPVQVFWQSTGR.T
2589	1	2	13/42	0.1831	0.1831	3.2526	UPSP:PARC_HUMAN	5	R.SGEALWLIPPQAYLNVEKDEGI
2881	1	3	33/84	0.4124	0.4124	4.8590	UPSP:PARC_HUMAN	5	R.SIFQPYISGPSLLLPTIVTTPR.R
2633	1	2	21/30	0.4106	0.4106	4.2923	UPSP:PARC_HUMAN	5	R.SLVGGPSAELLLDLER.V
1335	1	2	18/26	0.4121	0.4121	3.2879	UPSP:PARC_HUMAN	5	R.SPAPSPVLPSSSLR.N
2911	1	3	29/56	0.3227	0.3227	3.4478	UPSP:PARC_HUMAN	6	R.THQPINIPFFDVFLR.H
2248	1	2	10/16	0.0766	0.0766	2.1461	UPSP:PARC_HUMAN	5	R.TILM*M*LLNR.Y
1677	1	2	18/26	0.4394	0.4394	4.2665	UPSP:PARC_HUMAN	6	R.VGLQSPSVEAWEAK.G
2174	1	3	42/96	0.4362	0.4362	5.8289	UPSP:PARC_HUMAN	5	R.YIDQQIQGGLIGGAPGVEM*LG
2518	1	2	24/44	0.6084	0.6084	5.1031	UPSP:COA1_HUMAN	4	K.IASSIVAQTAGIPTLPWSGSGLR
1169	1	2	17/20	0.3587	0.3587	3.4775	UPSP:COA1_HUMAN	2	K.LLETESFQM*NR.I
2988	1	3	36/84	0.3926	0.3926	5.8721	UPSP:COA1_HUMAN	2	K.TLRDPSLPLLELQDIMTSVSGR.
2048	1	2	19/24	0.3977	0.3977	4.0295	UPSP:COA1_HUMAN	11	R.DFTVASPAEFVTR.F
2677	1	2	14/22	0.2809	0.2809	2.3326	UPSP:COA1_HUMAN	4	R.EGLPLM*VFANWR.G
2468	1	2	21/30	0.2462	0.2462	4.8216	UPSP:COA1_HUMAN	2	R.EVFFM*NTQSIVQLVQR.Y
2287	1	2	28/38	0.4931	0.4931	5.5083	UPSP:COA1_HUMAN	5	R.FIIGSVSEDNSEDEISNLVK.L
2531	1	2	20/36	0.6004	0.6004	4.9353	UPSP:COA1_HUMAN	3	R.FQAQSLGTTYIYDIPEM*FR.Q
1762	1	2	15/20	0.1824	0.1824	2.7586	UPSP:COA1_HUMAN	8	R.FVVM*VTPEDLK.A
2674	1	3	30/88	0.4627	0.4627	4.8288	UPSP:COA1_HUMAN	2	R.GQVLPAHTLLNTVDVELIYEGV
1437	1	2	15/24	0.2002	0.2002	2.4976	UPSP:COA1_HUMAN	4	R.GSVLEPEGTVEIK.F
2583	1	2	19/26	0.3954	0.3954	4.0987	UPSP:COA1_HUMAN	4	R.IGSFGPQEDLLFLR.A
1730	1	2	16/20	0.2618	0.2618	3.2307	UPSP:COA1_HUMAN	4	R.ILNVPQELYEK.G
1849	1	2	21/26	0.4457	0.4457	3.7010	UPSP:COA1_HUMAN	4	R.LGGIPVGVVAVETR.T
2149	1	2	15/22	0.2176	0.2176	2.3478	UPSP:COA1_HUMAN	2	R.NSVSNFLHSLER.G
1703	1	2	22/28	0.4143	0.4143	4.4010	UPSP:COA1_HUMAN	2	R.QVQAEVPGSPIFVM*R.L
0894	1	2	10/12	0.0982	0.0982	1.8971	UPSP:COA1_HUMAN	3	R.VLQAELK.I
0589	1	2	13/18	0.1672	0.1672	2.4670	UPSP:BRE1A_HUMAN	7	K.ASQEDANEIK.S
0946	1	2	19/24	0.3138	0.3138	3.1436	UPSP:BRE1A_HUMAN	4	K.EHLLQSNIGTGEK.E
0796	1	2	13/14	0.1685	0.1685	2.9068	UPSP:BRE1A_HUMAN	8	K.LAEM*LDQR.Q
1451	1	2	15/24	0.2550	0.2550	2.6223	UPSP:BRE1A_HUMAN	8	K.LGGVSSTEELDIR.T
1732	1	2	21/32	0.3728	0.3728	5.6487	UPSP:BRE1A_HUMAN	4	K.LLKEEKEELADQVLTLK.T
2208	1	2	21/30	0.4566	0.4566	4.5050	UPSP:BRE1A_HUMAN	15	K.LRTEVIQLEDTLAQVR.K
2353	1	2	15/28	0.1177	0.1177	3.0928	UPSP:BRE1A_HUMAN	5	K.RYDLEQGLGDLLTER.K
1182	1	2	16/20	0.2993	0.2993	3.6372	UPSP:BRE1A_HUMAN	4	K.TQVDAQLQVVR.K
1342	1	2	13/20	0.1326	0.1326	2.4132	UPSP:BRE1A_HUMAN	4	R.AVEEQIEYLQK.K
0899	1	2	13/14	0.3036	0.3036	2.7533	UPSP:BRE1A_HUMAN	8	R.HLAEVLER.V
1704	1	2	22/34	0.5526	0.5526	5.1598	UPSP:BRE1A_HUMAN	8	R.IEFEQTLAANEQAGPINR.E
1576	1	2	21/24	0.3696	0.3696	3.5760	UPSP:BRE1A_HUMAN	8	R.QATDDASLLIVNR.Y
2185	1	2	21/26	0.2503	0.2503	4.6004	UPSP:BRE1A_HUMAN	15	R.TEVIQLEDTLAQVR.K
2666	1	2	21/26	0.3495	0.3495	5.0179	UPSP:BRE1A_HUMAN	5	R.YDLEQGLGDLLTER.K
0492	1	2	21/26	0.4249	0.4249	4.4292	UPSP:P53_HUMAN	30	K.KGEPHHELPPGSTK.R
1741	1	2	22/26	0.4959	0.4959	4.1969	UPSP:P53_HUMAN	24	K.KKPLDGEYFTLQIR.G
1931	1	2	20/24	0.2864	0.2864	3.8880	UPSP:P53_HUMAN	24	K.KPLDGEYFTLQIR.G
0594	1	2	20/26	0.2153	0.2153	3.4733	UPSP:P53_HUMAN	28	K.RALPNNTSSSPQPK.K
1047	1	2	12/16	0.2469	0.2469	1.9706	UPSP:P53_HUMAN	27	K.TYQGSYGFR.L
0647	1	2	19/24	0.3882	0.3882	2.5403	UPSP:P53_HUMAN	28	R.ALPNNTSSSPQPK.K
1368	1	2	14/16	0.1126	0.1126	2.4803	UPSP:P53_HUMAN	25	R.ELNEALELK.D
0284	1	3	24/56	0.2531	0.2531	3.3097	UPSP:P53_HUMAN	30	R.KKGEPHHELPPGSTK.R
1027	1	2	17/18	0.2428	0.2428	3.2598	UPSP:P53_HUMAN	26	R.LGFLHSGTAK.S
2534	1	2	22/36	0.3437	0.3437	4.6797	UPSP:P53_HUMAN	27	R.RPILTIITLEDSSGNLLGR.N
0474	1	2	13/14	0.1311	0.1311	3.5412	UPSP:P53_HUMAN	30	R.RTEEENLR.K
0565	1	2	11/12	0.0199	0.0199	2.2577	UPSP:P53_HUMAN	30	R.TEEENLR.K
0933	1	1	8/12	0.1539	0.1539	2.0828	UPSP:P53_HUMAN	36	R.VEYLDDR.N
1643	1	2	18/22	0.3568	0.3568	3.3499	UPSP:BRE1B_HUMAN	8	K.AVEAAQLAEDLK.V

1665	1	3	31/92	0.2686	0.2686	4.1513	UPSP:BRE1B_HUMAN	5	K.KEELVPSEEDFQGITPGAQGPS
1648	1	3	29/64	0.2997	0.2997	5.1328	UPSP:BRE1B_HUMAN	8	K.LLREEKDELGEQVLGLK.S
1043	1	2	13/18	0.2125	0.2125	2.6100	UPSP:BRE1B_HUMAN	6	K.LQAELQGAVR.T
1351	1	2	19/22	0.3180	0.3180	4.0733	UPSP:BRE1B_HUMAN	7	K.SQVDAQLLTVQK.L
1406	1	2	13/18	0.1601	0.1601	2.2790	UPSP:BRE1B_HUMAN	5	K.TTTTLIEPIR.L
1728	1	2	21/30	0.4381	0.4381	3.7319	UPSP:BRE1B_HUMAN	6	R.EGPSLGPPPVASALSR.A
1503	1	2	18/20	0.3797	0.3797	3.7795	UPSP:BRE1B_HUMAN	4	R.ISLEYSELQDK.V
2268	1	3	33/76	0.3968	0.3968	5.2969	UPSP:BRE1B_HUMAN	8	R.KVEVYADADEILQEEIKEYK.A
1341	1	2	16/20	0.2731	0.2731	3.3607	UPSP:BRE1B_HUMAN	4	R.LQDLATQLQEK.H
1653	1	2	18/24	0.2330	0.2330	2.7766	UPSP:BRE1B_HUMAN	4	R.QATDDATLLIVNR.Y
2389	1	2	18/32	0.2824	0.2824	3.2441	UPSP:MCCA_HUMAN	3	K.AVNYVGAGTVEFIMDSK.H
1994	1	2	13/22	0.3402	0.3402	2.2949	UPSP:MCCA_HUMAN	3	K.EGSIEIDIPVPK.Y
1774	1	3	36/68	0.5230	0.5230	5.9785	UPSP:MCCA_HUMAN	3	K.IPLSQEEITLQGHAFEAR.I
1280	1	2	17/28	0.2422	0.2422	3.2982	UPSP:MCCA_HUMAN	2	K.KLGVQTVAVYSEADR.N
2188	1	2	21/28	0.4840	0.4840	4.2179	UPSP:MCCA_HUMAN	2	K.QEGIIFIGPPPSAIR.D
1428	1	3	28/92	0.2136	0.2136	3.8117	UPSP:MCCA_HUMAN	3	K.YLSSVSSQETQGGPLAPM*TGT
2318	1	3	38/92	0.4524	0.4524	5.3943	UPSP:MCCA_HUMAN	3	R.IYAEDPSNNFMPVAGPLVHLST
2233	1	3	28/84	0.3082	0.3082	4.2630	UPSP:MCCA_HUMAN	3	R.LQVEHPVTEM*ITGTDLVEWQ
2540	1	3	28/84	0.3397	0.3397	3.8825	UPSP:MCCA_HUMAN	3	R.LQVEHPVTEMITGTDLVEWQL
1363	1	2	15/24	0.2854	0.2854	3.2955	UPSP:MCCA_HUMAN	2	R.SEQEFQEQLESAR.R
2110	1	2	21/24	0.4837	0.4837	4.7499	UPSP:MCCC2_HUMAN	_	K.AFYGDTLVTGFAR.I
1144	1	2	12/18	0.1855	0.1855	2.0368	UPSP:MCCC2_HUMAN	1	K.GGAYYPVTVK.K
1483	1	2	18/30	0.4174	0.4174	3.8587	UPSP:MCCC2_HUMAN	1	K.QFSSADEAALKEPIIK.K
1805	1	2	11/12	0.2501	0.2501	2.3661	UPSP:MCCC2_HUMAN	1	K.TDFGIFR.M
2275	1	2	11/16	0.0573	0.0573	2.2997	UPSP:MCCC2_HUMAN	_	R.FLYIWPNAR.I
2587	1	2	26/40	0.6509	0.6509	5.2690	UPSP:MCCC2_HUMAN	_	R.IFGYPVGIVGNNGVLFSESAK.K
2156	1	2	26/34	0.4899	0.4899	5.6998	UPSP:MCCC2_HUMAN	1	R.ISVM*GGEQAANVLATITK.D
2457	1	2	23/34	0.4895	0.4895	5.7545	UPSP:MCCC2_HUMAN	1	R.ISVMGGEQAANVLATITK.D
1784	1	2	20/24	0.4001	0.4001	4.2929	UPSP:MCCC2_HUMAN	1	R.VWDDGIIDPADTR.L
2107	1	2	21/44	0.5074	0.5074	3.4224	UPSP:PYC_HUMAN	2	K.ASPSPTDPVVPAVPIGPPPAGFR
2355	1	2	16/28	0.4873	0.4873	3.6100	UPSP:PYC_HUMAN	2	K.DFTATFGPLDSLNTR.L
1518	1	2	22/26	0.4145	0.4145	4.7832	UPSP:PYC_HUMAN	2	R.AEAEAQAEELSFPR.S
0882	1	2	17/18	0.0863	0.0863	2.0370	UPSP:PYC_HUMAN	2	R.DAHQSLLATR.V
2760	1	2	18/24	0.4290	0.4290	3.1472	UPSP:PYC_HUMAN	1	R.ELIPNIPFQM*LLR.G
2904	1	2	15/24	0.2529	0.2529	2.5493	UPSP:PYC_HUMAN	1	R.ELIPNIPFQMLLR.G
1315	1	2	14/18	0.1215	0.1215	1.9974	UPSP:PYC_HUMAN	2	R.FIGPSPEVVR.K
1729	1	2	20/32	0.4933	0.4933	4.4194	UPSP:PYC_HUMAN	2	R.GANAVGYTNYPDNVVFK.F
2777	1	3	26/84	0.3297	0.3297	3.5389	UPSP:PYC_HUMAN	2	R.SVVEFLQGYIGVPHGGFPEPFR.
1630	1	2	17/18	0.3594	0.3594	3.5652	UPSP:FBXW8_HUMAN	1	K.ILVYSLEAGR.R
2270	1	2	19/30	0.3907	0.3907	3.3251	UPSP:FBXW8_HUMAN	1	K.IVSGGEEGLVSVWDYR.M
2025	1	2	19/22	0.3387	0.3387	3.2658	UPSP:FBXW8_HUMAN	1	K.LVQYLEIVPETR.R
2019	1	2	18/20	0.3763	0.3763	3.3645	UPSP:FBXW8_HUMAN	5	K.VIAEDEVLWYR.L
2045	1	2	22/28	0.2990	0.2990	4.4527	UPSP:FBXW8_HUMAN	_	R.EGAGGGEQLVDQLIR.D
1074	1	2	18/20	0.2797	0.2797	3.3050	UPSP:FBXW8_HUMAN	1	R.NADLDSFTTHR.R
1462	1	2	15/26	0.2783	0.2783	3.3211	UPSP:FBXW8_HUMAN	1	R.SGNIALSLSAHQLR.V
2232	1	2	21/26	0.4587	0.4587	4.1030	UPSP:PCCB_HUMAN	6	K.DTSYLFITGPDVVK.S
1883	1	2	19/42	0.0397	0.0397	2.8668	UPSP:PCCB_HUMAN	5	K.IM*DQAITVGAPVIGLNDSGGA
1061	1	2	21/30	0.4198	0.4198	4.9663	UPSP:PCCB_HUMAN	6	K.SVTNEDVTQEELGGAK.T
2839	1	2	24/32	0.5356	0.5356	5.8119	UPSP:PCCB_HUMAN	6	R.IQEGVESLAGYADIFLR.N
1894	1	2	20/22	0.2659	0.2659	3.8074	UPSP:HSP7C_HUMAN	4	K.DAGTIAGLNVLR.I
1840	1	2	21/26	0.4605	0.4605	3.7092	UPSP:HSP7C_HUMAN	4	K.SFYPEEVSSM*VLTK.M
2716	1	2	25/44	0.5891	0.5891	5.4449	UPSP:HSP7C_HUMAN	4	K.SINPDEAVAYGAAVQAAILSG
1900	1	2	23/34	0.3810	0.3810	4.2849	UPSP:HSP7C_HUMAN	4	K.TVTNAVVTVPAYFNDSQR.Q
2049	1	2	22/28	0.5366	0.5366	4.5951	UPSP:PCCA_HUMAN	2	K.VVEEAPSIFLDAETR.R
1667	1	3	25/52	0.2501	0.2501	3.0198	UPSP:PCCA_HUMAN	3	R.LSQYQEPLHLPGVR.V
1600	1	2	15/20	0.3208	0.3208	3.1775	UPSP:PCCA_HUMAN	4	R.M*ADALDNYVIR.G
2003	1	3	27/60	0.0005	0.0005	3.4776	UPSP:PCCA_HUMAN	5	R.M*PVIKPDIANWELSVK.L
2076	1	2	15/26	0.2393	0.2393	2.3419	UPSP:HS70L_HUMAN	15	K.AFYPEEISSM*VLTK.L
2094	1	2	15/22	0.2268	0.2268	2.8966	UPSP:HS70L_HUMAN	15	K.DAGVIAGLNVLR.I
2698	1	2	13/44	0.4081	0.4081	2.9671	UPSP:HS70L_HUMAN	15	K.SINPDEAVAYGAAVQAAILM*
1459	1	2	18/24	0.4309	0.4309	3.5593	UPSP:HS70L_HUMAN	27	R.TTPSYVAFTDTER.L
2058	1	2	19/26	0.4227	0.4227	3.8537	UPSP:NUCL_HUMAN	7	K.GFGFVDFNSEEDAK.A
2857	1	2	25/42	0.4911	0.4911	5.9118	UPSP:NUCL_HUMAN	6	K.TLVLSNLSYSATEETLQEVFEK
2792	1	2	23/40	0.5780	0.5780	5.2820	UPSP:NUCL_HUMAN	5	K.VEGTEPTTAFNLFVGNLNFNK.
1938	1	2	16/18	0.2873	0.2873	3.6891	UPSP:RLA0_HUMAN	6	K.IIQLLDDYPK.C
0706	1	2	14/20	0.3366	0.3366	2.4440	UPSP:RLA0_HUMAN	6	R.GHLENNPALEK.L

2472	1	2	21/32	0.5052	0.5052	5.5043	UPSP:RLA0_HUMAN	7	R.VLALSVETDYTFPLAEK.V
2266	1	2	22/30	0.4192	0.4192	5.0809	UPTR:Q59FY4_HUMAN	3	K.IIQQAGQVWFPDSAFK.T
1904	1	2	19/26	0.1171	0.1171	2.4930	UPTR:Q59FY4_HUMAN	3	R.GGSWVVIDSSINPR.H
1901	1	2	22/34	0.2880	0.2880	3.5641	UPTR:Q59FY4_HUMAN	3	R.TVELSIPADPANLDSEAK.I
0798	1	2	11/16	0.1888	0.1888	2.3522	UPSP:NPM_HUMAN	18	K.GPSSVEDIK.A
2689	1	2	23/40	0.6239	0.6239	4.6132	UPSP:NPM_HUMAN	20	K.M*SVQPTVSLGGFEITPPVVLR.
2533	1	2	19/26	0.2970	0.2970	3.9932	UPSP:NPM_HUMAN	11	R.M*TDQEAIQDLWQWR.K
0640	1	2	19/24	0.3327	0.3327	4.6123	UPSP:DECR_HUMAN	2	K.ATAEQISSQTGNK.V
1482	1	2	16/24	0.1156	0.1156	2.5887	UPSP:DECR_HUMAN	2	K.VAFITGGGTGLGK.G
1692	1	3	27/84	0.3211	0.3211	3.6761	UPSP:DECR_HUMAN	2	K.VAGHPNIVINNAAGNFISPTER.
0922	1	2	13/14	0.1143	0.1143	2.3145	GP:M66870_1	_	R.FDGTVEVK.D
0982	1	2	22/28	0.3936	0.3936	3.9822	GP:M66870_1	_	R.GASQNIIPSSTGAAK.A
1924	1	2	21/26	0.4195	0.4195	4.1809	GP:M66870_1	_	R.VPTPNVSVVDLTVR.L
1890	1	3	29/72	0.2315	0.2315	3.4752	UPTR:Q53EN9_HUMAN	6	K.HSSDASSLLPQNILSQTSR.H
2360	1	2	19/38	0.4075	0.4075	4.3969	UPTR:Q53EN9_HUMAN	6	K.INEVLTAAVTQASLQSIIHK.F
0789	1	2	12/20	0.1137	0.1137	2.0907	UPTR:Q53EN9_HUMAN	2	R.RGDSQPYQALK.Y
2773	1	2	22/30	0.2324	0.2324	3.9932	UPSP:TCPE_HUMAN	3	K.LGFAGLVQEISFGTTK.D
2875	1	2	18/32	0.4420	0.4420	4.0095	UPSP:TCPE_HUMAN	3	R.WVGGPEIELIAIATGGR.I
0949	1	2	18/30	0.3473	0.3473	3.7787	UPSP:HSP72_HUMAN	8	K.STAGDTHLGGEDFDNR.M
1903	1	3	23/44	0.2137	0.2137	4.0527	UPSP:HSP72_HUMAN	7	R.ARFEELNADLFR.G
2811	1	2	18/24	0.4370	0.4370	3.8651	UPSP:RS2_HUMAN	7	K.SLEEIYLFSLPIK.E
2211	1	2	14/20	0.4109	0.4109	2.9228	UPSP:RS2_HUMAN	8	K.TYSYLTPDLWK.E
2548	1	3	32/84	0.3385	0.3385	5.3337	UPSP:HNRPU_HUMAN	9	K.EKPYFPIPEEYTFIQNVPLEDR.V
1986	1	2	25/30	0.0119	0.0119	5.1561	UPSP:GRP78_HUMAN	18	R.IINEPTAAAIAYGLDK.R
2405	1	2	22/28	0.3826	0.3826	4.6294	UPSP:TBA6_HUMAN	8	R.AVFVDLEPTVIDEVR.T
1893	1	3	32/60	0.4303	0.4303	4.5035	UPSP:RL4_HUMAN	3	K.APIRPDIVNFVHTNLR.K
1720	1	3	28/96	0.3142	0.3142	4.3642	UPTR:Q96FW6_HUMAN	8	R.ISTPQTNTVPIKPLISTPPVSSQPI
1432	1	2	18/24	0.3204	0.3204	4.2556	UPSP:COA2_HUMAN	9	K.EASFEYLQNEGER.L
2022	1	2	23/30	0.4136	0.4136	4.2360	UPSP:HSP71_HUMAN	9	R.IINEPTAAAIAYGLDR.T
1101	1	2	22/28	0.2959	0.2959	4.0354	GP:BC022805_1	1	K.GTAAAAAAAAAAAAK.V
2083	1	3	25/84	0.3031	0.3031	4.0322	UPTR:Q9NQS8_HUMAN	5	K.FVDGLM*IHSGDPVNYYVDTA
2203	1	2	20/36	0.4141	0.4141	4.0315	UPSP:RL9_HUMAN	1	K.TILSNQTVDIPENVDITLK.G
1713	1	2	20/26	0.3716	0.3716	3.9831	UPTR:Q5VVC8_HUMAN	8	K.VLEQLTGQTPVFSK.A
2027	1	2	22/34	0.4536	0.4536	3.9612	UPSP:TCPH_HUMAN	3	K.VQGGALEDSQLVAGVAFK.K
2381	1	2	21/28	0.3859	0.3859	3.8553	UPSP:IF2B_HUMAN	4	K.DASDDLDDLNFFNQK.K
2385	1	2	18/26	0.4023	0.4023	3.8533	UPSP:RL7_HUMAN	7	R.IVEPYIAWGYPNLK.S
2000	1	3	27/76	0.2011	0.2011	3.8384	UPSP:MUC2_HUMAN	1	R.EVGQYLVVESSTGIIVIWDK.R
2563	1	3	31/108	0.3080	0.3080	3.7911	UPTR:Q7Z561_HUMAN	3	K.M*ADHYVPVPGGPNNNNYAN
2073	1	3	28/68	0.3904	0.3904	3.4785	UPSP:EFG2_HUMAN	3	R.VNLIDTPGHVDFTLEVER.C
1616	1	2	16/20	0.1198	0.1198	3.4583	UPSP:G3PT_HUMAN	4	K.AGIALNDNFVK.L
2255	1	2	16/24	0.4014	0.4014	3.3386	UPTR:Q8TCX0_HUMAN	_	K.GQSLQDPFLNALR.R
2253	1	2	17/24	0.2877	0.2877	3.1118	UPSP:RL18_HUMAN	3	K.ILTFDQLALDSPK.G

Supplementary Table S3. Primer sequences for ChIP-qPCR analysis of the *p21* locus.

Position	Forward Primer	Reverse Primer
P21-1 in Figure3	AGCAGGCTGTGGCTCTGATT	CAAAATAGCCACCAGCCTCTTCT
P21-2 in Figure3	CTGTCCTCCCCGAGGTCA	ACATCTCAGGCTGCTCAGAGTCT
P21-3 in Figure3<br>*P21*-1 in Figure6	TATATCAGGGCCGCGCTG	GGCTCCACAAGGAACTGACTTC
P21-4 in Figure3<br>*P21-2 in Figure6*	CCAGGAAGGGCGAGGAAA	GGGACCGATCCTAGACGAACTT
P21-5 in Figure3<br>*P21-3 in Figure6*	AGCCGGAGTGGAAGCAGA	AGTGATGAGTCAGTTTCCTGCAAG
P21-6 in Figure3<br>*P21-4 in Figure6*	CCAGGGCTGCGATTAGGAA	GTGTCCCTCAGGGTGTGAAT
P21-5 in Figure6	ACCCCCTTTCCTGGACACTC	GTCACCCTGCCCAACCTTAG
P21-7 in Figure3<br>*P21-6 in Figure6*	CCTCCCACAATGCTGAATATACAG	AGTCACTAAGAATCATTTATTGAGCAC

Supplementary Table S4. Primer sequences for ChIP-qPCR analysis of the *PUMA* locus.

Position	Forward Primer	Reverse Primer
PUMA-1 in Figure S4	TCAGTGTGTGTGTCCGACTGTC	GGCAGGGCCTAGCCCA
PUMA-2 in Figure S4<br>*PUMA*-1 in Figure S7	GCCCTCCTGAAGGAAGCC	GCCTGCACTCCTGTCACCT
PUMA-3 in Figure S4<br>*PUMA*-2 in Figure S7	GTGGAGTCTGCGGCTCCT	CCAATAACCGGCTGTTGC
PUMA-4 in Figure S4<br>*PUMA*-3 in Figure S7	GTTGCTGACAGTTGGTGATGA	ACATGCCATCAGACCTCAACA
PUMA-5 in Figure S4<br>*PUMA*-4 in Figure S7	AGGTGCTGCTCCGCCA	CCCTCTGCCTCTCCAAGGTC
PUMA-6 in Figure S4<br>*PUMA*-5 in Figure S7	GAAGTCCCACCTGCCGTCTA	GGACGACCTCAACGCACAGT

Supplementary Table S5. Primer sequences for RT-PCR analysis of *p21*mature and pre-mRNA expression.

Gene	Forward Primer	Reverse Primer
p21 pre-mRNA	TGAGGTGACACAGCAAAGCC	GCCATTAGCGCATCACAGTC
*p21*mature mRNA	CCTCCCACAATGCTGAATATACAG	AGTCACTAAGAATCATTTATTGAGCAC
GAPDH	ACCCACTCCTCCACCTTTGA	CTGTTGCTGTAGCCAAATTCGT

Supplementary Table S6. Primer sequences for RT-PCR analysis of *PUMA* mature and pre-mRNA expression.

Gene	Forward Primer	Reverse Primer
PUMA pre-mRNA	AGGATAGGTTGCAGTCCATC	CTCATCCAGGACATGAGAC
PUMA mature-mRNA	CAATCTCATCATGGGACTCC	GTCCAGTATGCTACATGGTG
GAPDH	ACCCACTCCTCCACCTTTGA	CTGTTGCTGTAGCCAAATTCGT
